# Isolable Geminal Bisgermenolates: A New Synthon in Organometallic Chemistry

**DOI:** 10.1002/anie.202111636

**Published:** 2021-09-28

**Authors:** Manfred Drusgala, Philipp Frühwirt, Gabriel Glotz, Katharina Hogrefe, Ana Torvisco, Roland C. Fischer, H. Martin R. Wilkening, Anne‐Marie Kelterer, Georg Gescheidt, Michael Haas

**Affiliations:** ^1^ Institute of Inorganic Chemistry Graz University of Technology Stremayrgasse 9/IV 8010 Graz Austria; ^2^ Institute of Physical and Theoretical Chemistry Graz University of Technology Stremayrgasse 9/II 8010 Graz Austria; ^3^ Institute for Chemistry and Technology of Materials Graz University of Technology Stremayrgasse 9/III 8010 Graz Austria

**Keywords:** acylgermanes, bisenolates, germenolates, photoinitiators, SET reaction

## Abstract

We have synthesized the first isolable geminal bisenolates L_2_K_2_Ge[(CO)R]_2_ (*R*=2,4,6‐trimethylphenyl (**2 a**,**b**), L=THF for (**2 a**) or [18]‐crown‐6 for (**2 b**)), a new synthon for the synthesis of organometallic reagents. The formation of these derivatives was confirmed by NMR spectroscopy and X‐ray crystallographic analysis. The UV/Vis spectra of these anions show three distinct bands, which were assigned by DFT calculations. The efficiency of **2 a**,**b** to serve as new building block in macromolecular chemistry is demonstrated by the reactions with two different types of electrophiles (acid chlorides and alkyl halides). In all cases the salt metathesis reaction gave rise to novel Ge‐based photoinitiators in good yields.

## Graphical abstract

A convenient method to obtain geminal bisenolates L_2_K_2_Ge[(CO)R]_2_ (*R*=2,4,6‐trimethylphenyl (**1 a**,**b**), L=THF for (**1 a**) or [18]‐crown‐6 for (**1 b**)), a new synthon for the synthesis of organometallic reagents, is reported. Selected examples of electrophiles were used to display the efficiency of **1 a**,**b** as new building blocks in organometallic chemistry.

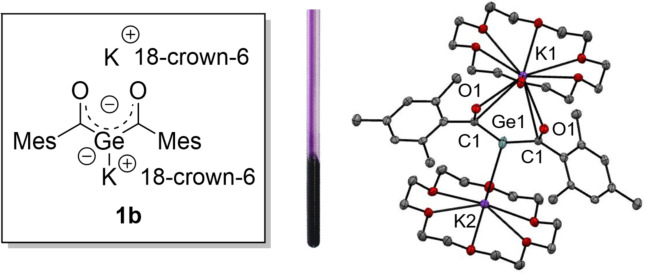

The chemistry of metal enolates continues to be a comprehensively researched field in contemporary organic chemistry.[^1^, ^2^] Furthermore, the classical aldol reaction is one of the most important biosynthetic tools for life on earth.^[3]^ In this context a geminal bisenolate surrogate was introduced as powerful organic substrate for the regioselective α,α‐difunctionalization adjacent to a ketone.^[4]^ This surrogate bypasses the use of α‐diazo carbonyl compounds, which are state‐of‐the‐art reagents for such reactions.^[5]^ Here a replacement is highly desirable owing to safety concerns and limited functional group compatibility of the latter. There are, however, no reports on the targeted isolation of geminal bisenolates published so far.

Recently heavier Group 14 enolates (HG 14 enolates) of silicon and germanium were found to be key intermediates during the synthesis of photoinitiators (PIs), as well as during the formation of complex silicon frameworks.[^6^, ^7^, ^8^]

The latest investigation concerning germenolates was the synthesis and characterization of potassium‐tris(2,4,6‐trimethylbenzoyl)germenolate (**1**), as a new building block for high‐potential PIs.[^9^, ^10^]

While the chemistry towards tetraacylgermanes has already been thoroughly investigated,[^7^, ^8^] the class of triacylgermenolates is still in the early stages of development. Owing to the importance of the latter, as highly promising building block for germanium‐based PIs, we continued to examine this particular compound class.

During the course of these examinations, a new hitherto unpublished and yet undescribed species was found. Upon addition of 2.0 equivalents of elemental potassium to a solution of **1** dissolved in THF, we established a highly selective electron transfer reaction leading to the elimination of a mesitoyl substituent. Hence, the dianionic species **2** is formed. This remarkable finding represents the first ever synthesized geminal bisenolate so far and opens up a new highly reactive building block in main group chemistry. Looking at the literature only a handful geminal germyl dianions are reported.

The groups of Mochida, Satgé, and Tokitoh pioneered in the synthesis of 1,1‐dimetalogermane derivatives of the type Ar_2_GeM_2_ (compound **3 a**, Scheme 1) by the metalation of the corresponding hydrides with alkali metals, ^*t*^BuLi or lithium naphthalenide.^[11]^ Vyazankin et al. generated Et_2_GeLi_2_ (compound **3 b**, Scheme 2) by the reaction of (Me_3_Si)Et_2_GeLi with Me_3_SiLi.^[12]^ However, due to their thermal instability, these compounds were not isolable and were only identified by trapping reactions. Sekiguchi and Lee used silylated precursor molecules to perform either a reductive cycloreversion or a direct reductive dehalogenation to synthesize isolable 1,1‐dilithiogermane derivatives (compound **4**, Scheme 1).^[13]^ These silyl‐substituted geminal dianions are isolable, room‐temperature stable, and fully characterizable compounds, which led to their importance as synthetic building blocks in modern main group chemistry.

**Scheme 1:**
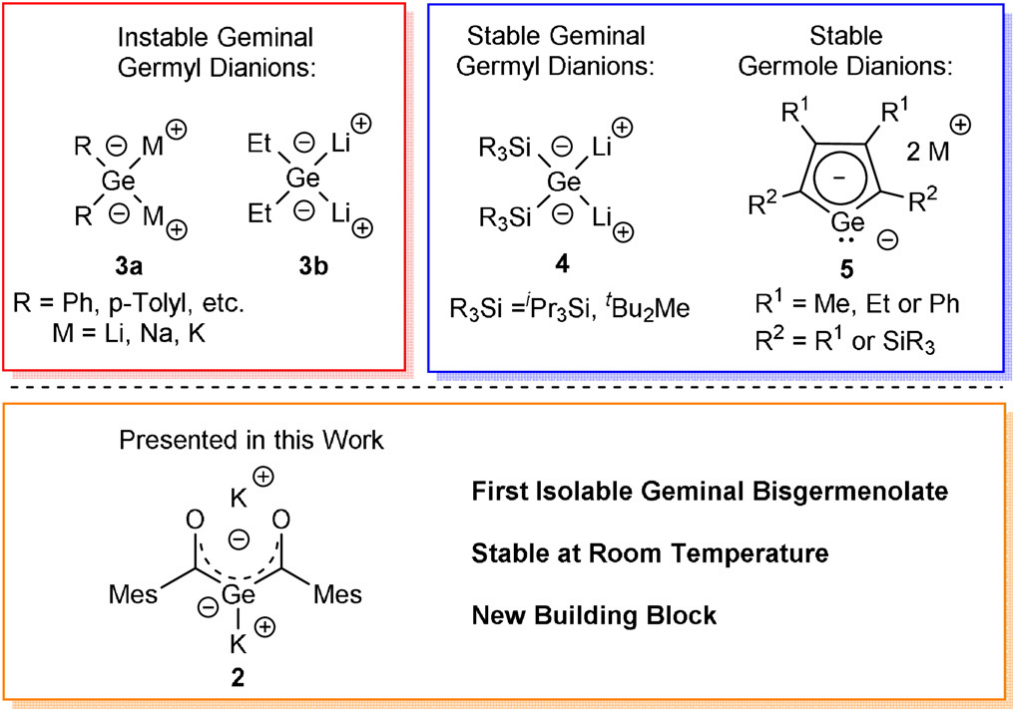
Types of known geminal dianionic germanium compounds **3**–**4** and germole dianions **5**. New isolable geminal bisgermenolate **2** as new building block.

**Scheme 2:**
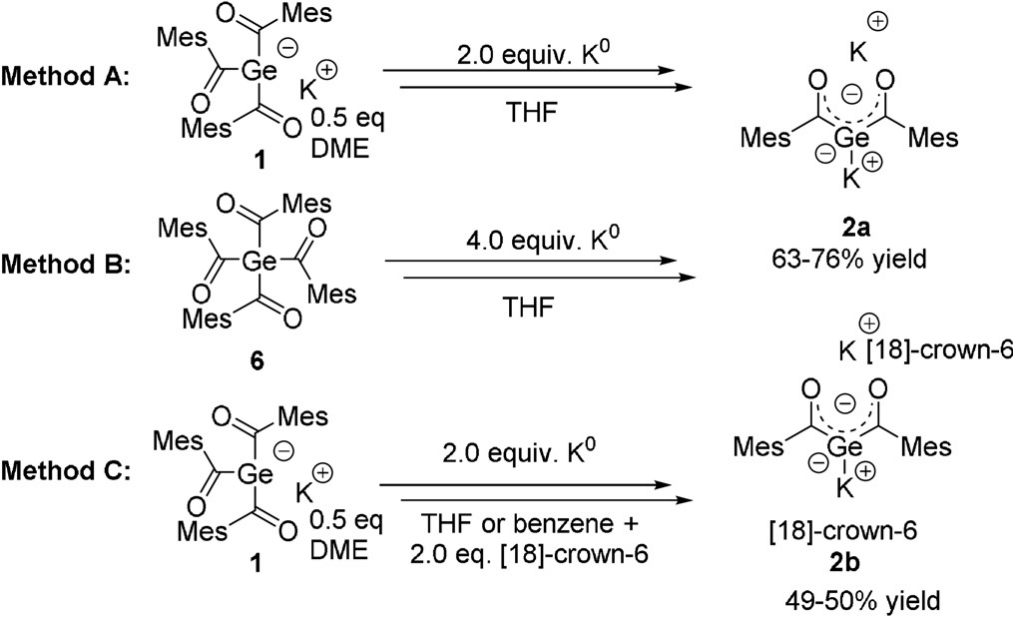
Synthesis of **2 a**,**b**.

Another important germanium‐based dianion are germole dianions. Since West et al. reported on the first characterized delocalized germole dianion,^[14]^ considerable progress has been made during the last two decades. Especially the groups of Tilley,^[15]^ Boudjouk^[16]^ and Müller^[17]^ synthesized a variety of germole dianions and discussed their chemistry (compound **5**, Scheme 1) The rare number of geminal dianions available and hence the undeniable interest in these derivatives drove our motivation to contribute to this field. Thereby our intention was the design of a reasonable preparative‐scale approach for the synthetically highly attractive geminal bisgermenolate.

The synthesis of **2** can be performed by the use of two different starting materials and two different ligand systems, with equal reaction mechanism for all methods. These reactions resulted in the clean formation of air and moisture sensitive geminal bisgermenolate **2 a**,**b** in good yields (Scheme 2). The details of the SET mechanism is depicted on the next page. During the reaction, **2 a**,**b** precipitated at room temperature from the respective used solvent to give purple crystals. After filtration, the crystals can be stored at room temperature in the absence of air even for prolonged periods. Analytical data are consistent with the proposed structures, as both derivatives show very similar ^13^C chemical shifts for the carbonyl C‐atom for **2 a** *δ*=274.7 ppm and for **2 b** *δ*=279.4 ppm, which is characteristic for carbonyl groups directly linked to a negative charged germanium atom. On the basis of the very low solubility of **2 a**,**b** in all standard solvents for NMR spectroscopy (highest solubility was observed in [D_8_]THF with 5 mg mL^−1^ for **2 a** and 12 mg mL^−1^ for **2 b**) we also performed MAS‐NMR spectroscopy to ensure the purity of these compounds. **2 a**,**b** show very similar shifts in the ^1^H and ^13^C MAS‐NMR. Moreover, for **2 b** we also observed the shift for the carbonyl group at *δ*=278.4 ppm. The results are presented in the Supporting Information.

Compound **2** can be described by two resonance structures **2′** and **2′′**, shown in Figure 1. **2′** represents the geminal dianion and **2′′** represents the bisgermenolate. Moreover, **2** can also be described as **2′′′**, which is an isomer of **2′** and **2′′**.

**Figure 1:**
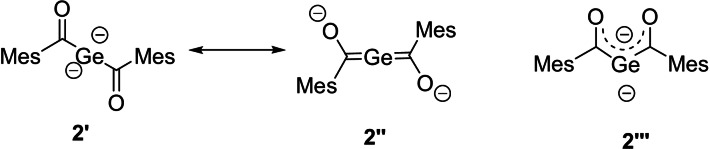
Possible resonance and isomeric structures of **2**.

Derivative **2 b** afforded crystals of sufficient quality for single crystal X‐ray crystallography. The molecular structure is depicted in Figure 2. Compound **2 b** crystallizes together with two molecules of [18]‐crown‐6 in the orthorhombic space group *Pnma* containing four molecules per unit cell. Both acyl groups are stabilized by one potassium atom, while the central Ge‐atom is stabilized by the other potassium atom.

**Figure 2:**
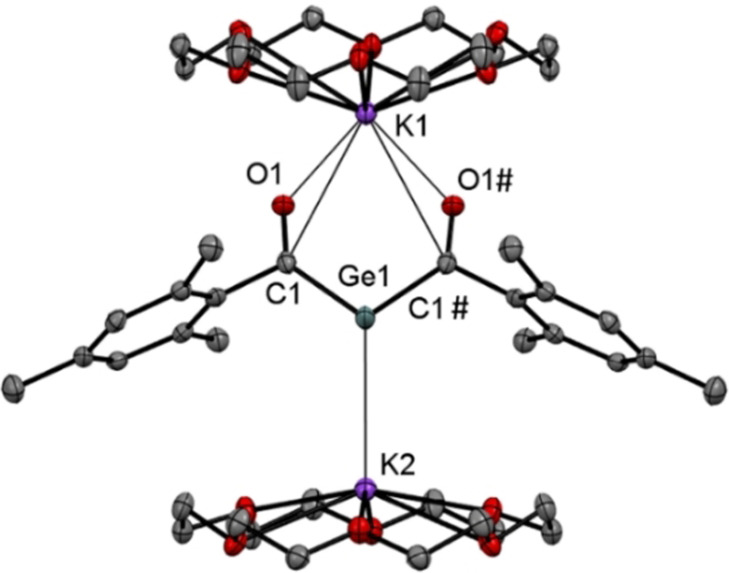
ORTEP representation for compound **2 b** (1:2 adducts with [18]‐crown‐6). Ellipsoids are set at 50 % probability. Hydrogen atoms and solvent molecules are omitted for clarity. Selected bond lengths [pm] and angles [°] with estimated standard deviations: Ge1–C1 198.4(3), C1–O1 125.2(4), Ge1–K1 396.6(12), Ge1–K2 333.6(11), O1–K1 264.8(2); C1‐Ge1‐C1# 103.64(19).

On the basis of the observed structural features, **2 b** adopts in the solid‐state structure **2′′′**. This is also supported by DFT calculations. The negative charge is mainly confined in the C(O)GeC(O) moiety of **2 b** (Figure 3) explaining the rather tight binding of one K^+^ counterion towards the two carbonyl oxygen atoms (distance: ca. 265 pm). Owing to the concentration of −0.58 charge units at the Ge center, the second K^+^ counterion resides below it at a distance of 334 pm, with the two K cations being slightly tilted along the *y*‐axis.

**Figure 3:**
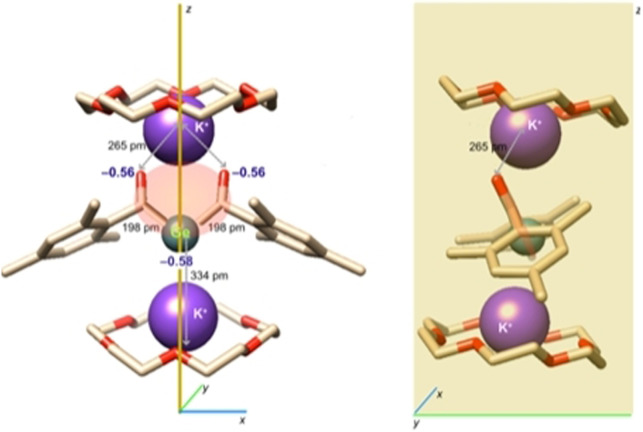
DFT calculated geometry of **2 b** including selected charges and bond lengths. Left: view along the *x*,*z* plane, right: view along the *y*,*z* plane. H‐Atoms are not displayed for clarity. The molecular moiety carrying 85 % of the negative charge is marked in salmon.

The Ge−C(O) bonds of 198 pm are slightly shorter compared to values of literature‐known germenolates (210‐206 pm),^[18]^ but significantly longer then values found for germenes (177–186 pm),^[19]^ indicating electron delocalization across the C(O)−Ge−C(O) fragment.

To study the reaction mechanism for the formation of **2 a**,**b**, we used a combination of UV/Vis and EPR spectroscopy. A solution of tetraacylgermane **6** in dry THF was prepared in a special EPR tube allowing an in situ reduction at a potassium mirror (see the Supporting Information for details).

The solution was gradually reduced until the violet color of the dianion appeared. EPR and UV/Vis spectra were recorded after each contact of the solution with the potassium mirror. During the procedure, the characteristic absorption band of tris(mesitoyl)germenolate (*λ*_max_=427 nm^[7]^) was detected via UV/Vis spectroscopy. Further reduction led to the characteristic UV/Vis spectrum of the dianion **2 a** (dark violet solution; see Figure 4). The detailed results are presented in the Supporting Information.

**Figure 4:**
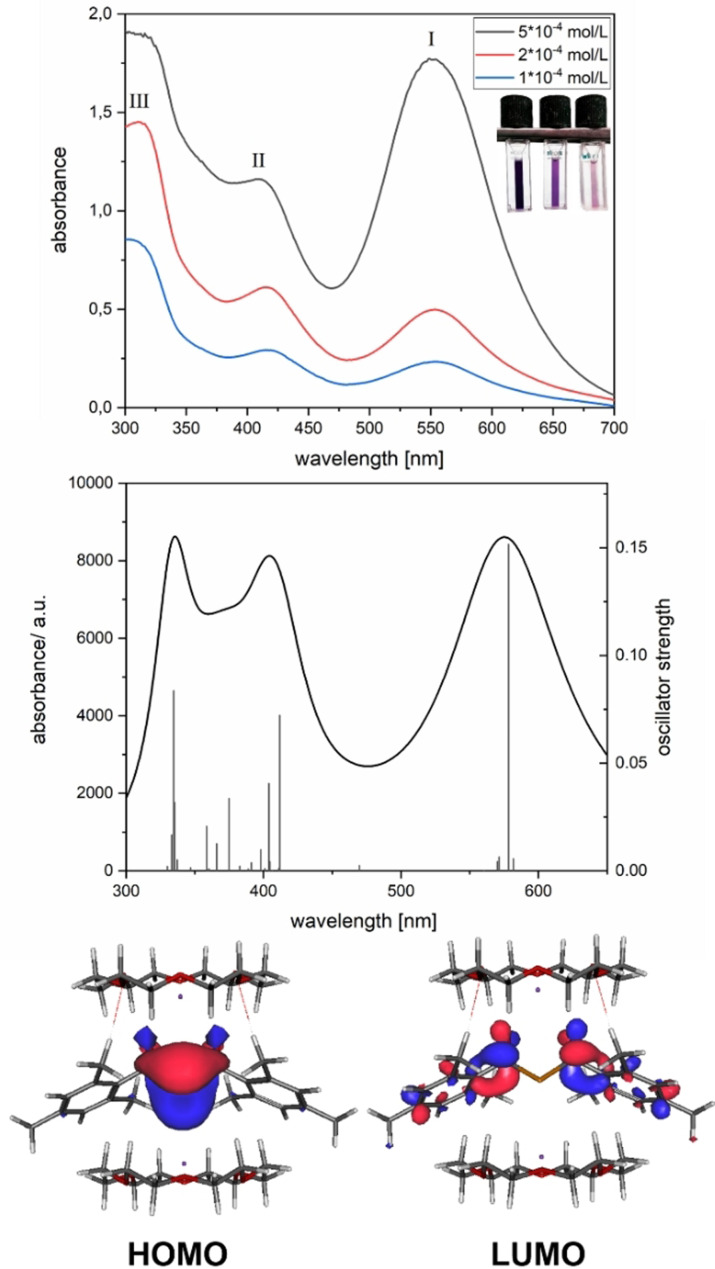
Measured UV/Vis spectra and calculated contributions of **2 b** in THF. Vertical transitions are marked as lines with their respective oscillator strengths (right axis). The orbitals involved in the first bands are presented in the lower part.

Furthermore, we investigated a THF solution of **1** after exposure to metallic potassium and could obtain an EPR spectrum (shown in the Supporting Information). The spectrum is unresolved likely based on many small and unresolved coupling constants of the protons of the mesityl substituent. Instead, another reaction mixture (**6** + potassium + [18]‐crown‐6 in benzene) gave a rather distinct EPR spectrum (Supporting Information). In our prior publication on the characterization of the germenolate **1**, an almost identical EPR spectrum was obtained, however, the reduction was performed with KO*t*Bu. We tentatively assigned the spectrum to a follow‐up product derived from **1** and the *tert*‐butoxy radical.^[9]^ The new experimental results contradict our first assignment and rather suggest, that both reducing agents lead to the same intermediate or by‐product. As reducing agent, the use of elemental potassium is essential. With other metals (i.e. lithium or sodium) or bases (i.e. KO*t*Bu) no formation of **2** was observed.

As reported recently, the germenolate **1** is formed by a germa‐acyloin condensation.^[9]^ During the formation of **2**, this reaction sequence is repeated for the second acyl‐group. Here, once more a radical anion is formed by a SET reaction (Scheme 3). Hence, two ion‐paired radical anions undergo a germa‐acyloin condensation followed by a scission of the diketone‐moiety. After elimination of the diketone, the bisgermenolate **2** is formed. A second SET reaction probably leads to the formation of a diradical, which undergoes a complex and uncharacterized degradation sequence. The formation of **2 a** by method B, depicted in Scheme 2, performs both SET reactions consecutively, starting from the tetraacylgermane **6**. Herein, the formation of the germenolate **1** is instantly followed by the conversion to the bisgermenolate **2**. In the case of method C, the bisacylgermenolate **2** is stabilized additionally by two equivalents of [18]‐crown‐6.

**Scheme 3:**
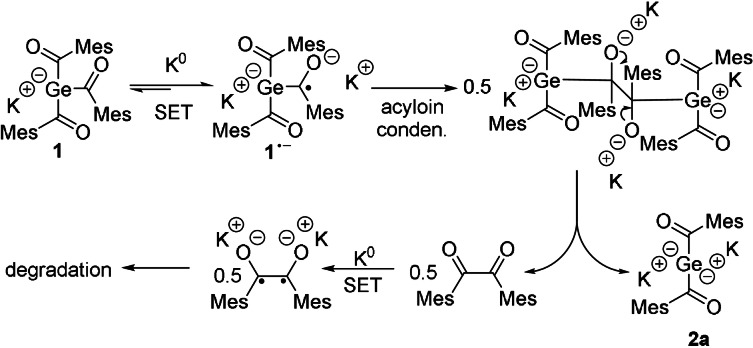
SET reaction of **1** with K^0^ (Method A).

THF was used as a solvent to determine the charge transfer behavior for the longest wavelength absorption bands.^[20]^ Figure 4 depicts the measured and calculated UV/Vis spectra of **2 b** in THF together with their calculated frontier Kohn–Sham orbitals for the HOMO and LUMO (the UV/Vis spectrum of **2 a** can be found in the Supporting Information).

The geminal bisgermenolate **2 b** exhibits three intense absorption bands with *λ*_max_=553 nm (band I), 416 nm (band II) and 311 nm (band III). Moreover, the calculated data show a reasonable agreement with the measured absorption bands. The band at around 570 nm is dominated by a HOMO→LUMO transition. The HOMO has significant coefficients at the Ge‐atom. The LUMO reveals a symmetric electron distribution over both mesitoyl moieties with the negative charge residing mainly at the central part of **2 b** (see Figure 4). The absorption centered at 416 nm consists of two main contributions from HOMO→LUMO+6 and HOMO→LUMO+7 transitions (for the orbital representations and the detailed interpretation of the spectrum see Supporting Information).

This dianionic species represents an optimal and highly promising building block for the formation of new, hitherto not synthesizable acylgermanes and related species. In order to test our new precursor system, we reacted **2 a**,**b** with selected examples of electrophiles. As suitable electrophiles we choose aryl‐ as well as alkyl‐substituted acid chlorides and two haloalkanes. All these reactions resulted in the clean formation of a variety of new acylgermanes in good yields (Scheme 4). A significant correlation between the used method and the yield of the expected product was not observed. Analytical and spectroscopic data obtained for **7 a**–**g** are consistent with the proposed structures. NMR spectra and detailed assignments are provided in the Experimental Section in the Supporting Information.

**Scheme 4:**
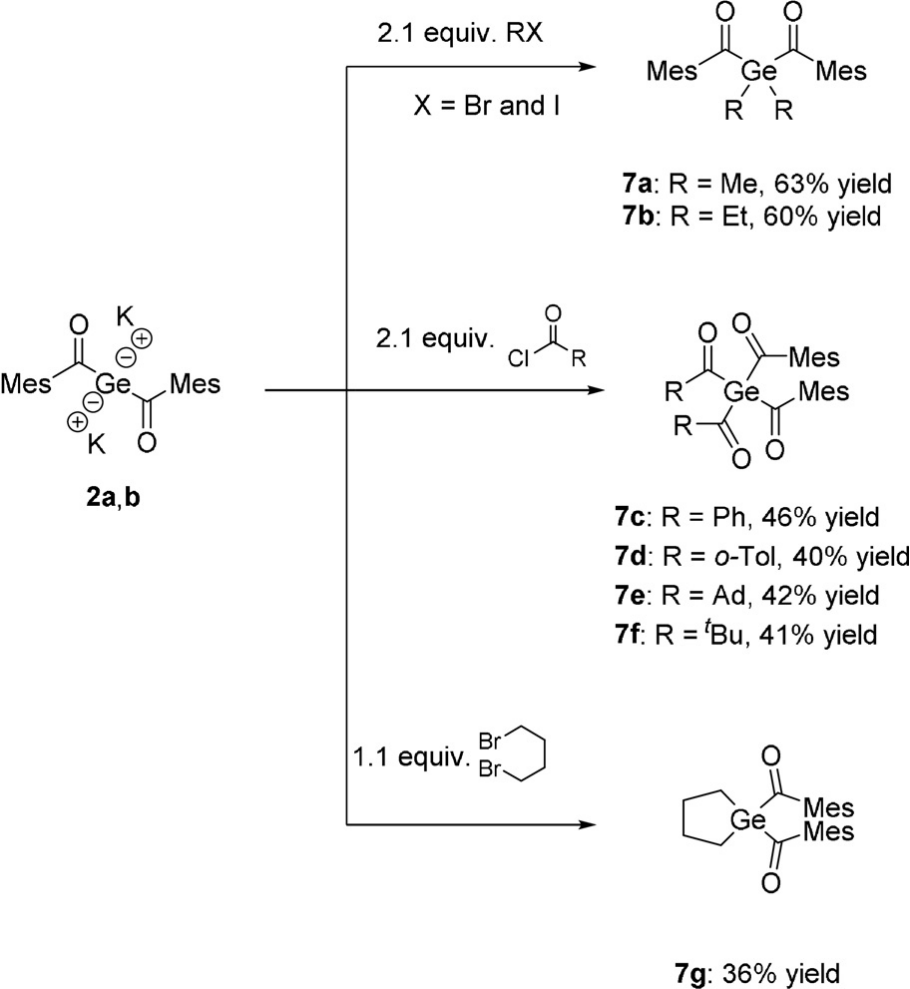
Reactivity of **2 a**,**b** with selected examples of electrophiles.

The UV/Vis spectra of **7 a**–**g** (Figure 5) revealed broad absorption bands centered at around 390 nm for **7 a**–**d** and at around 360 nm for **7 e**–**g**, which can be allocated for the n‐π* transition. This band is responsible for the photo‐induced cleavage of the Ge−C bond. In close analogy to previous observations, the mixed functionality of these derivatives leads to broadening of their absorption bands, which is highly beneficial for their application as larger variety of wavelengths can be used to initiate photopolymerization.

**Figure 5:**
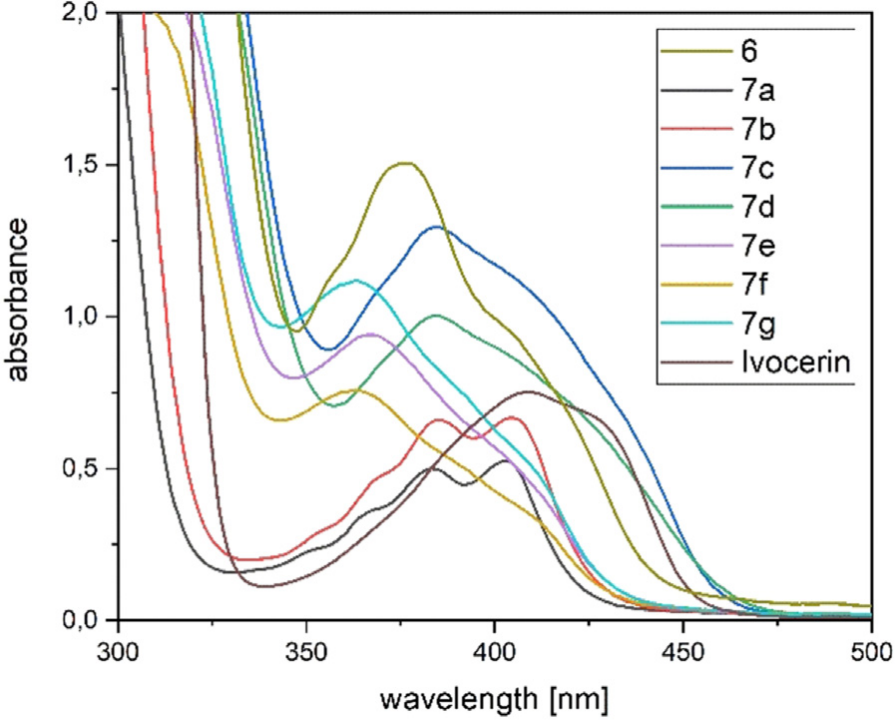
Absorption spectra of synthesized compounds **7 a**–**g** compared with the commercially available Ivocerin and tetra(mesitoyl)‐germane **6** at a concentration of 1×10^−3^ M in dichloromethane.

All derivatives show very similar ^13^C chemical shifts for the carbonyl C‐atom between *δ*=223.9 and 243.8 ppm, which is characteristic for carbonyl groups directly linked to a germanium atom. Crystals suitable for single‐crystal X‐ray diffraction analysis were obtained for compounds **7 a**, **7 f**, and **7 g**. As a representative example, the molecular structure of **7 g** is depicted in Figure 6 (see the Supporting Information for remaining structures). Structural data are in accordance with literature values of other acyl germanium compounds.[^7^, ^8^, ^18^, ^21^, ^22^]

**Figure 6:**
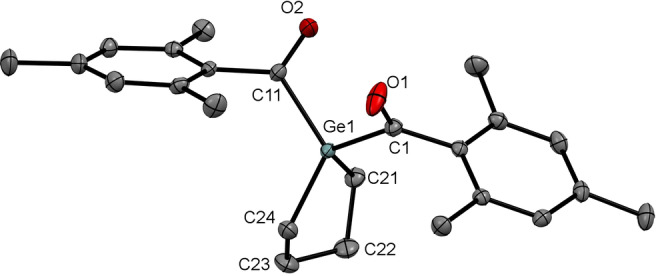
ORTEP representation for compound **7 g**. Ellipsoids are set at 50 % probability. Hydrogen atoms and solvent molecules are omitted for clarity. Selected bond lengths [Å] with estimated standard deviations: Ge1–C1 2.0057(15), C1–O1 1.212(2), C11–O2 1.216(2), Ge1–C11 2.0037(15), Ge1–C21 1.9624(16), Ge1–C24 1.9644(16).

To explore if the acylgermanes synthesized via the procedure shown in Scheme 3 are suitable photoinitiators, we performed photo CIDNP experiments^[23]^ with **7 g** and **7 f**. The corresponding ^1^H‐NMR/CIDNP spectra in the presence of butyl acrylate clearly reveal the growth of polymer chains with Ge‐substituted end groups based on the expected α‐cleavage of Ge−C(O) bonds upon n–π* excitation via intersystem crossing (see Supporting Information).[^7^, ^10^, ^22^]

In conclusion, we have introduced a synthetic strategy for the hitherto unknown geminal bisenolates L_2_K_2_Ge[(CO)R]_2_ (*R*=2,4,6‐trimethylphenyl (**2 a**,**b**), L=THF for (**2 a**) or [18]‐crown‐6 for (**2 b**)), which represents a new synthon for the synthesis of organometallic reagents. Furthermore, the formation of these derivatives was confirmed by NMR spectroscopy and X‐ray crystallographic analysis.^[24]^

Moreover, the efficiency of **2 a**,**b** to serve as new building block in macromolecular chemistry is demonstrated by the reactions with two different types of electrophiles (acid chlorides and alkyl halides). In all cases the salt metathesis reaction gave rise to potential novel Ge‐based photoinitiators in good yields. Further studies to probe the scope of this chemistry are currently in progress.

## Conflict of interest

The authors declare no conflict of interest.

## Supporting information

As a service to our authors and readers, this journal provides supporting information supplied by the authors. Such materials are peer reviewed and may be re‐organized for online delivery, but are not copy‐edited or typeset. Technical support issues arising from supporting information (other than missing files) should be addressed to the authors.

Supporting Information

## References

[1a] D. Stolz , U. Kazmaier in The Chemistry of Metal Enolates, Vol. 1 (Eds.: Z. Rappoport , J. Zabicky ), Wiley-VCH, Hoboken, 2009, pp. 355–411;

[1b] D. Caine in Comprehensive Organic Synthesis, Vol. 3 (Eds.: B. M. Trost , I. Fleming ), Pergamon, Oxford, 1991, pp. 1–63.

[2a] N. Khiar , I. Fernández , A. Alcudia , M. V. García , R. Recio in Carbohydrates. Tools for stereoselective synthesis (Ed.: M. M. K. Boysen ), Wiley-VCH, Weinheim, 2013, pp. 47–63;

[2b] D. Vargová , I. Némethová , K. Plevová , R. Šebesta , ACS Catal. 2019, 9, 3104–3143;

[2c] B. M. Trost , J. S. Tracy , Acc. Chem. Res. 2020, 53, 1568–1579. 32692147 10.1021/acs.accounts.0c00285

[3a] A. Szekrenyi , X. Garrabou , T. Parella , J. Joglar , J. Bujons , P. Clapés , Nat. Chem. 2015, 7, 724–729; 26291944 10.1038/nchem.2321

[3b] M. Rueping , Angew. Chem. Int. Ed. 2006, 45, 1838–1840; 10.1002/anie.200504053 16521102

[3c] S. Fusz , A. Eisenführ , S. G. Srivatsan , A. Heckel , M. Famulok , Chem. Biol. 2005, 12, 941–950; 16125106 10.1016/j.chembiol.2005.06.008

[3d] M. Braun in Modern Aldol Reactions, Vol. 1 (Ed.: R. Mahrwald ), Wiley-VCH, Weinheim, 2004, pp. 1–61.

[4] C. E. Iacono , T. C. Stephens , T. S. Rajan , G. Pattison , J. Am. Chem. Soc. 2018, 140, 2036–2040. 29381360 10.1021/jacs.7b12941

[5a] J. R. Denton , H. M. L. Davies , Org. Lett. 2009, 11, 787–790; 19146454 10.1021/ol802614j

[5b] J. Tao , R. Tran , G. K. Murphy , J. Am. Chem. Soc. 2013, 135, 16312–16315; 24152071 10.1021/ja408678p

[5c] E. Emer , J. Twilton , M. Tredwell , S. Calderwood , T. L. Collier , B. Liégault , M. Taillefer , V. Gouverneur , Org. Lett. 2014, 16, 6004–6007; 25379614 10.1021/ol5030184

[5d] M. J. James , P. O'Brien , R. J. K. Taylor , W. P. Unsworth , Angew. Chem. Int. Ed. 2016, 55, 9671–9675; 10.1002/anie.201605337 27435296

[5e] W. Yuan , L. Eriksson , K. J. Szabó , Angew. Chem. Int. Ed. 2016, 55, 8410–8415; 10.1002/anie.201602137 PMC5089581 27219856

[6a] M. Leypold , L. Schuh , R. Fischer , A. Torvisco , M. Flock , H. Stueger , M. Haas , Angew. Chem. Int. Ed. 2017, 56, 8089–8093; 10.1002/anie.201701862 28481426

[6b] J. Radebner , A. Eibel , M. Leypold , N. Jungwirth , T. Pickl , A. Torvisco , R. Fischer , U. K. Fischer , N. Moszner , G. Gescheidt , H. Stueger , M. Haas , Chem. Eur. J. 2018, 24, 8281–8285. 29709089 10.1002/chem.201801622 PMC6032833

[7] J. Radebner , A. Eibel , M. Leypold , C. Gorsche , L. Schuh , R. Fischer , A. Torvisco , D. Neshchadin , R. Geier , N. Moszner , R. Liska , G. Gescheidt , M. Haas , H. Stueger , Angew. Chem. Int. Ed. 2017, 56, 3103–3107; 10.1002/anie.201611686 28156043

[8] J. Radebner , M. Leypold , A. Eibel , J. Maier , L. Schuh , A. Torvisco , R. Fischer , N. Moszner , G. Gescheidt , H. Stueger , M. Haas , Organometallics 2017, 36, 3624–3632.

[9] P. Frühwirt , A. Knoechl , M. Pillinger , S. M. Müller , P. T. Wasdin , R. C. Fischer , J. Radebner , A. Torvisco , N. Moszner , A.-M. Kelterer , T. Griesser , G. Gescheidt , M. Haas , Inorg. Chem. 2020, 59, 15204–15217. 32993291 10.1021/acs.inorgchem.0c02181 PMC7581296

[10] S. D. Püschmann , P. Frühwirt , M. Pillinger , A. Knöchl , M. Mikusch , J. Radebner , A. Torvisco , R. C. Fischer , N. Moszner , G. Gescheidt , M. Haas , Chem. Eur. J. 2021, 27, 3338–3347. 33034922 10.1002/chem.202004342 PMC7898609

[11a] K. Mochida , N. Matsushige , M. Hamashima , Bull. Chem. Soc. Jpn. 1985, 58, 1443;

[11b] A. Castel , P. Rivière , J. Satgé , Y.-H. Ko , J. Organomet. Chem. 1988, 342, C1–C4;

[11c] A. Castel , P. Riviere , J. Satge , H. Y. Ko , Organometallics 1990, 9, 205–210;

[11d] A. Castel , P. Rivière , J. Satgé , D. Desor , J. Organomet. Chem. 1992, 433, 49–61;

[11e] N. Tokitoh , K. Hatano , T. Sadahiro , R. Okazaki , Chem. Lett. 1999, 28, 931–932.

[12] D. A. Bravo-Zhivotovskii , S. D. Pigarev , O. A. Vyazankina , N. S. Vyazankin , Zh. Obshch. Khim. 1987, 57, 2644–2645.

[13a] A. Sekiguchi , R. Izumi , S. Ihara , M. Ichinohe , V. Y. Lee , Angew. Chem. Int. Ed. 2002, 41, 1598–1600; 10.1002/1521-3773(20020503)41:9<1598::aid-anie1598>3.0.co;2-8 19750678

[13b] V. Y. Lee , K. McNeice , Y. Ito , A. Sekiguchi , Chem. Commun. 2011, 47, 3272–3274. 10.1039/c0cc05415a 21286637

[14] R. West , H. Sohn , D. R. Powell , T. Müller , Y. Apeloig , Angew. Chem. Int. Ed. Engl. 1996, 35, 1002–1004;

[15] W. P. Freeman , T. D. Tilley , L. M. Liable-Sands , A. L. Rheingold , J. Am. Chem. Soc. 1996, 118, 10457–10468.

[16a] S.-B. Choi , P. Boudjouk , J.-H. Hong , Organometallics 1999, 18, 2919–2921;

[16b] S.-B. Choi , P. Boudjouk , K. Qin , Organometallics 2000, 19, 1806–1809.

[17a] Z. Dong , C. R. W. Reinhold , M. Schmidtmann , T. Müller , Organometallics 2018, 37, 4736–4736;

[17b] Z. Dong , L. Albers , T. Müller , Acc. Chem. Res. 2020, 53, 532–543. 32031772 10.1021/acs.accounts.9b00636

[18] M. Haas , M. Leypold , D. Schnalzer , A. Torvisco , H. Stueger , Organometallics 2015, 34, 5291–5297.

[19a] V. Y. Lee , A. Sekiguchi , Organometallics 2004, 23, 2822–2834;

[19b] K. M. Baines , W. G. Stibbs , Coord. Chem. Rev. 1995, 145, 157–200.

[20] C. Reichardt , T. Welton , Solvents and Solvent Effects in Organic Chemistry, 4th ed., Wiley-VCH, Weinheim, 2011, pp. 1–718.

[21a] M. Haas , Chem. Eur. J. 2019, 25, 15218–15227; 31325194 10.1002/chem.201902307 PMC6916596

[21b] T. Wiesner , M. Leypold , A. Steinmaurer , D. Schnalzer , R. C. Fischer , A. Torvisco , M. Haas , Organometallics 2020, 39, 2878–2887. 32801424 10.1021/acs.organomet.0c00385 PMC7422643

[22] H. Stueger , M. Haas , J. Radebner , A. Eibel , G. Gescheidt , Chem. Eur. J. 2018, 24, 8258–8267. 29356151 10.1002/chem.201705567 PMC6032850

[23] A. Yurkovskaya , O. Morozova , G. Gescheidt in Encyclopedia of radicals in chemistry, biology and materials (Eds.: C. Chatgilialoglu , A. Studer ), Wiley, Chichester, 2012.

[24] Deposition Numbers 2089189 (for **2b**), 2089190 (for **7a**), 2089191 (for **7f**), and 2089192 (for **7g**) contain the supplementary crystallographic data for this paper. These data are provided free of charge by the joint Cambridge Crystallographic Data Centre and Fachinformationszentrum Karlsruhe Access Structures service www.ccdc.cam.ac.uk/structures.

