# Supplementary material for: Isolable Geminal Bisgermenolates: A New Synthon in Organometallic Chemistry
Source: Angew Chem Int Ed Engl. 2021 Sep 28;60(44):23646–50. doi: 10.1002/anie.202111636 (PMC8596710; doi:10.1002/anie.202111636)
Supplement: Supplementary file 1 — Supporting Information [file ANIE-60-23646-s001.pdf]

Angewandte Chemie
Eine Zeitschrift der Gesellschaft Deutscher Chemiker

Supporting Information

Isolable Geminal Bisgermenolates: A New Synthon in Organometallic Chemistry

*Manfred Drusgala, Philipp Frühwirt, Gabriel Glotz, Katharina Hogrefe, Ana Torvisco, Roland C. Fischer, H. Martin R. Wilkening, Anne-Marie Kelterer, Georg Gescheidt, and Michael Haas**

anie_202111636_sm_miscellaneous_information.pdf

Table of Content

Experimental Procedures 2
General Considerations 2
Reaction of 1 with K^o (Method A) 2
Reaction of 6 with K^o (Method B) 2
Reaction of 1 with K^o and [18]-crown-6 in THF (Method C) 2
Reaction of 1 with K^o and [18]-crown-6 in benzene (Method C) 3
Reaction of 2a with MeI 3
Reaction of 2a with EtBr 3
Synthesis of 7c 4
Synthesis of 7d 4
Synthesis of 7e 4
Synthesis of 7f 5
Synthesis of 7g 5
DFT Calculations 6
Mechanistic Investigations 11
CIDNP Experiments 14
MAS NMR Experiments 18
NMR-Spectroscopy 19
Figure S10: ^{1}H- and ^{13}C-NMR spectra of $K_2Ge(C(O)Mes)_2$ (2a) 19
Figure S11: MAS ^{1}H- and ^{13}C-NMR spectra of $K_2Ge(C(O)Mes)_2$ (2a) 20
Figure S12: ^{1}H- and ^{13}C-NMR spectra of $K_2Ge(C(O)Mes)_2$*2 18-crown-6 (2b) 21
Figure S13: MAS ^{1}H- and ^{13}C-NMR spectra of $K_2Ge(C(O)Mes)_2$*2 18-crown-6 (2b) 22
Figure S14: ^{1}H- and ^{13}C-NMR spectra of $(Me)_2Ge(C(O)Mes)_2$ (7a) 23
Figure S15: ^{1}H- and ^{13}C-NMR spectra of $(Et)_2Ge(C(O)Mes)_2$ (7b) 24
Figure S16: ^{1}H- and ^{13}C-NMR spectra of $(PhC(O))_2Ge(C(O)Mes)_2$ (7c) 25
Figure S17: ^{1}H- and ^{13}C-NMR spectra of $(o\text{-}TolC(O))_2Ge(C(O)Mes)_2$ (7d) 26
Figure S18: ^{1}H- and ^{13}C-NMR spectra of $(AdC(O))_2Ge(C(O)Mes)_2$ (7e) 27
Figure S19: ^{1}H- and ^{13}C-NMR spectra of $(t\text{Bu}C(O))_2Ge(C(O)Mes)_2$ (7f) 28
Figure S20: ^{1}H- and ^{13}C-NMR spectra of $(C_4H_8)Ge(C(O)Mes)_2$ (7g) 29
UV-Vis Spectroscopy 30
Figure S21: UV-Vis Spectrum of 2a with three different concentrations. Measured in THF. 30
X-ray Crystallography 31
Crystallographic Table 32
Figure S22: ORTEP representation for compound 7a 33
Figure S23: ORTEP representation for compound 7f 33
References 34

Experimental Procedures

General Considerations

All experiments were performed under a nitrogen atmosphere using standard Schlenk techniques. Solvents were dried using a column solvent purification system.[1] Me_3SiCl (≤99%), $GeCl_4$ (>99,99%), KO*t*Bu (>98%), ClCO(Mes) (98 %), [18]-crown-6 (99%), potassium (98%), MeI (>99%), EtBr (≤99), ClCO(Ph) (≤99%), ClCO(o-Tol) (98 %), ClCO($C_{10}H_{15}$) (95%), ClCO(C_4H_9) (98%), 1,4-dibromobutane (98%), THF-d_8 (99.5 atom% D), toluene-d_8 (99.0 atom% D), acetonitrile-d_3 (≤99.8 atom% D), C_6D_6 (99.5 atom%, D) and $CDCl_3$ (99.8 atom% D) were used without any further purification. Tetrakis(trimethylsilyl)germane[2], tetraacylgermane **6** and FCO(Mes) was prepared according to published procedures.[3]
^{1}H- and ^{13}C-NMR spectra were recorded on a Varian INOVA 300 spectrometer in C_6D_6, THF-d_8, toluene-d_8, acetonitrile-d_3 or $CDCl_3$ solutions and referenced versus TMS using the internal ^{2}H-lock signal of the solvent. HRMS spectra were run on a Kratos Profile mass spectrometer. Infrared spectra were obtained on a Bruker Alpha-P Diamond ATR Spectrometer from the solid sample. Melting points were determined using Stuart SMP50 apparatus and are uncorrected. Elemental analyses were carried out on a Hanau Vario Elementar EL apparatus. UV absorption spectra were recorded on a Perkin Elmer Lambda 5 spectrometer. NMR spectra under magic angle spinning (MAS) conditions were recorded on a 500 MHz Avance spectrometer (Bruker) with a nominal magnetic field of 11.7 T.

Reaction of 1 with K^o (Method A)
A flask was charged with 2.00 g potassium-tris(2,4,6-trimethylbenzoyl)germanide*0.5 DME (3.34 mmol; 1,00 eq.) and 0.26 g potassium (6.68 mmol; 2.00 eq.). Subsequently 12 ml THF was added. The suspension was stirred for 48 hours. The violet crystalline product was filtered off and washed with cold diethylether and benzene. Yield: 0.93 g (2.09 mmol; 63 %) of analytically pure **2a** as violet powder (note: **2a** contains 1 molecule of THF)

Reaction of 6 with K^o (Method B)
A flask was charged with 2.00 g tetrakis(2,4,6-trimethylbenzoyl)germane (3.02 mmol; 1.00 eq.) and 0.47 g potassium (12.10 mmol; 4.00 eq.). Subsequently 20 ml THF was added. The suspension was stirred for 48 hours. The violet crystalline product was filtered off and washed with cold diethylether and benzene. Yield: 1,02 g (2.29 mmol; 76 %) of analytically pure **2a** as violet powder (note: **2a** contains 1 molecule of THF).

2a: **mp:** 250-260°C (dec.). **Anal. Calc.** for $C_{24}H_{30}GeK_2O_3$: C, 55.72; H, 5.85 % **Found**: C, 56.01; H, 5.63 %. **^{13}C-NMR** (THF-d_8, TMS, ppm): 274.65 (*C*=O), 150.23 (Mes-*C4*), 135.34 (Mes-*C1*), 131.66 (Mes-*C2*), 128.77, 128.31 (Mes-*C3*), 21.30, 21.19, 20.12, 20.00 (Aryl-*C*H$_3$). **^{1}H-NMR** (THF- d_8, TMS, ppm): 6.60 (s, 4H, Mes-*H*), 2.20 (s, 12H, Mes-C*H*$_3$), 2.16 (s, 6H, Mes-C*H*$_3$). **UV-Vis:** λ [nm] (ε [L mol^{-1} cm^{-1}]) = 545 (3856), 419 (6325), 320 (18350). **IR** (neat): ν(C=O) = 1560.

Reaction of 1 with K^o and [18]-crown-6 in THF (Method C)
A flask was charged with 2.00 g potassium-tris(2,4,6-trimethylbenzoyl)germanide*0.5 DME (3.34 mmol; 1.00 eq.), 1.76 g [18]-crown-6 (6.68 mmol; 2.00 eq.) and 0.26 g potassium (6.68 mmol; 2.00 eq.). Subsequently 12 ml THF were added. The suspension was stirred for 48 hours. The violet crystalline product was filtered off and washed with cold *n*-pentane and benzene. Yield: 1,74 g (1.79 mmol; 54%) of analytically pure **2b** as violet powder.

Reaction of 1 with K^o and [18]-crown-6 in benzene (Method C)
A flask was charged with 2.00 g potassium-tris(2,4,6-trimethylbenzoyl)germanide*0.5 DME (3.34 mmol; 1.00 eq.), 1.77 g [18]-crown-6 (6.68 mmol; 2.00 eq.) and 0.26 g potassium (6.68 mmol; 2.00 eq.). Subsequently 12 ml benzene were added. The suspension was stirred for 48 hours. The violet crystalline product was filtered off and washed with cold *n*-pentane and benzene. Yield: 1,61 g (1.65 mmol; 50 %) of analytically pure **2b** as violet powder.

2b: mp: 110-130°C. **Anal. Calc.** for $C_{43}H_{65}GeK_2O_{14}$: C, 53.81; H, 7.14 % **Found**: C, 53.60; H, 7.24 %. **^{13}C-NMR** (THF-d_8, TMS, ppm): 279.60 (*C*=O), 154.66 (Mes-*C4*), 132.95 (Mes-*C1*), 131.90 (Mes-*C2*), 127.89 (Mes-*C3*), 71.43 ((-*C*H_2-*C*H_2-O-)$_6$), 23.24, 22.36, 21.13 (Aryl-*C*H_3). **^{1}H-NMR** (THF-d_8, TMS, ppm): 6.55 (s, 4H, Mes-*H*), 3.49 (s, 48H, (-C*H*$_2$-C*H*$_2$-O-)$_6$), 2.50 (s, 6H, Mes-C*H*$_3$), 2.14 (s, 12H, Mes-C*H*$_3$). **UV-Vis:** λ [nm] (ε [L mol^{-1} cm^{-1}]) = 553 (1502), 416 (3137), 311 (7360). **IR** (neat): ν(C=O) = 1577.

Reaction of 2a with MeI
0.22 ml iodomethane (3.51 mmol; 2.10 eq.) was added to a solution of **2a** at -70°C [prepared from 1.00 g (1.67 mmol; 1.00 eq.) **1** with 0,13 g (3.34 mmol; 2.00 eq.) potassium in 12ml THF]. Afterwards, the reaction mixture was brought to room temperature and stirred for another hour. The solution was added to 200 ml of saturated NH_4Cl solution and ice. After phase separation, three-fold washing of the aqueous phase with 100 ml of Et_2O, drying of the combined organic layers with Na_2SO_4 and evaporation of the solvents in vacuum, the product was purified via flash column chromatography (*n*-pentane/diethylether 3:1) to result a pale yellow crystalline solid. Yield: 0.42 g (1.06 mmol; 63 %) of analytically pure **7a**.

7a: mp: 80-82°C. **Anal. Calc.** for $C_{22}H_{28}GeO_2$: C, 66.54; H, 7.11 % **Found**: C, 66.67 H, 7.34 %. **^{13}C-NMR** ($CDCl_3$, TMS, ppm): 243.68 (*C*=O), 142.26 (Mes-*C4*), 138.74 (Mes-*C1*), 131.82 (Mes-*C2*), 128.83 (Mes-*C3*), 21.18, 18.91 (Aryl-*C*H_3), -2.54 (Ge-*C*H_3). **^{1}H-NMR** ($CDCl_3$, TMS, ppm): 6.79 (s, 4H, Mes-*H*), 2.27 (s, 6H, Mes-C*H*$_3$), 2.09 (s, 12H, Mes-C*H*$_3$), 0.51 (s, 6H, Ge-C*H*$_3$). **UV-Vis:** λ [nm] (ε [L mol^{-1} cm^{-1}]) = 403 (526), 383 (500), 364 (338), 350 (224). **IR** (neat): ν(C=O) = 1635, 1608.

Reaction of 2a with EtBr
0.23 ml bromoethane (3.05 mmol; 2.10 eq.) was added to a solution of **2a** at -70°C [prepared from 0.87 g (1.45 mmol; 1.00 eq.) **1** with 0,11 g (2.90 mmol; 2.00 eq.) potassium in 12ml THF]. Afterwards, the reaction mixture was brought to room temperature and stirred for another hour. The solution was added to 200 ml of saturated NH_4Cl solution and ice. After phase separation, three-fold washing of the aqueous phase with 100 ml of Et_2O, drying of the combined organic layers with Na_2SO_4 and evaporation of the solvents in vacuum, the product was recrystallized from acetone and isolated. Yield: 0.37 g (0.87 mmol; 60 %) of analytically pure **7b** as pale yellow crystalline solid.

7b: mp: 89-95 °C. **Anal. Calc.** for $C_{24}H_{32}GeO_2$: C, 67.80; H, 7.59 % **Found**: C 67.68; H 7.62, %. **^{13}C-NMR** ($CDCl_3$, TMS, ppm): 243.78 (*C*=O), 143.21 (Mes-*C4*), 138.72 (Mes-*C1*), 131.87 (Mes-*C2*), 128.90 (Mes-*C3*), 21.24, 18.94 (Aryl-*C*H_3), 8.78 (-CH_2-*C*H_3), 7.41 (Ge-*C*H2-). **^{1}H-NMR** ($CDCl_3$, TMS, ppm): 6.77 (s, 4H, Mes-*H*), 2.26 (s, 6H, Mes-C*H*$_3$), 2.10 (s, 12H, Mes-C*H*$_3$), 1.15 (q, ^{2}J = 7.7 Hz, 4H, Ge-C*H*$_2$), 1.00 (t, ^{2}J = 7.7 Hz, 6H, -CH_2-C*H*$_3$). **UV-Vis:** λ [nm] (ε [L mol^{-1} cm^{-1}]) = 405 (667), 385 (660), 367 (450), 354 (295). **IR** (neat): ν(C=O) = 1646, 1629, 1607. **HRMS** (MNa^+): calc. 449.1517, found: 449.1563.

Synthesis of 7c

2.94 ml benzoyl chloride (25.05 mmol; 5.00 eq.) was added to a solution of **2a** at -70°C [prepared from 3.00 g (5.01 mmol; 1.00 eq.) **1** with 0.39 g (10.01 mmol; 2.00 eq.) potassium in 15ml THF]. Afterwards, the reaction mixture was brought to room temperature and stirred for another hour. The solution was added to 200 ml of saturated NH_4Cl solution and ice. After phase separation, three-fold washing of the aqueous phase with 100 ml of Et_2O, drying of the combined organic layers with Na_2SO_4 and evaporation of the solvents in vacuum, the product was purified via flash column chromatography (toluene) to result a yellow oil. Yield: 1.32 g (2.29 mmol; 46 %) of analytically pure **7c**.

7c: Anal. Calc. for $C_{34}H_{32}GeO_4$: C, 70.74; H, 5.59 % **Found**: C 70.79; H 5.62, %. ^{13}**C-NMR** (C_6D_6, TMS, ppm): 231.80, 221.49 (*C*=O), 142.33 (Mes-*C4*), 140.80 (Ph-*C1*), 139.62 (Mes-*C1*), 133.60 (Ph-*C4*), 133.21 (Mes-*C2*), 129.41 (Ph-*C2*), 129.12 (Ph-*C3*), 128.81 (Mes-*C3*), 20.95, 19.43 (Aryl-*C*H_3). ^{1}**H-NMR** (C_6D_6, TMS, ppm): 7.95-7.92 (m, 4H, Aryl-*H*), 6.88 (m, 6H, Aryl-*H*), 6.29 (s, 4H, Mes-*H*), 2.28 (s, 12H, Mes-C*H3*), 1.86 (s, 6H, Mes-C*H3*). **UV-Vis:** λ [nm] (ε [L mol^{-1} cm^{-1}]) = 384 (1366), 261 (28200). **IR** (neat): ν(C=O) = 1643, 1631. **HRMS** (MNa^+): calc. 601.1418, found: 601.1481.

Synthesis of 7d

0.74 ml 2-methylbenzoyl chloride (5.65 mmol; 2.10 eq.) was added to a solution of **2a** at -70°C [prepared from 1.61 g (2.69 mmol; 1.00 eq.) **1** with 0.21 g (5.37 mmol; 2.00 eq.) potassium in 12ml THF]. Afterwards, the reaction mixture was brought to room temperature and stirred for another hour. The solution was added to 200 ml of saturated NH_4Cl solution and ice. After phase separation, three-fold washing of the aqueous phase with 100 ml of Et_2O, drying of the combined organic layers with Na_2SO_4 and evaporation of the solvents in vacuum, the product was purified via flash column chromatography (toluene/*n*-pentane 2:1) to result a yellow oil. Yield: 0.65 g (1.07 mmol; 40 %) of analytically pure **7d**.

7d: Anal. Calc. for $C_{36}H_{36}GeO_4$: C, 71.43; H, 5.99 % **Found**: C 71.25; H 5.87 %. ^{13}**C-NMR** ($CDCl_3$, TMS, ppm): 233.14, 223.88 (*C*=O), 141.71 (Mes-*C4*), 139.69 (Mes-*C1*), 139.62 (*o*-Tol-*C1*), 137.27 (*o*-Tol-*C2*), 133.47 (*o*-Tol-*C4*), 132.76 (*o*-Tol-*C3*), 131.85 (Mes-*C2*), 131.78 (*o*-Tol-*C6*), 128.86 (Mes-*C3*), 125.68 (*o*-Tol-*C5*), 21.17, 21.02, 19.24 (Aryl-*C*H_3). ^{1}**H-NMR** ($CDCl_3$, TMS, ppm): 7.64 (d, ^{2}J = 7.6 Hz, 2H, Ph-*H*), 7.23-7.07 (m, ^{2}J = 7.6 Hz, 8H, Ph-*H*), 6.57 (s, 4H, Mes-*H*), 2.44 (s, 6H, Ph-C*H3*), 2.16 (s, 6H, Mes-C*H*3), 2.16 (s, 12H, Mes-C*H*3). **UV-Vis:** λ [nm] (ε [L mol^{-1} cm^{-1}]) = 384 (1002). **IR** (neat): ν(C=O) = 1725, 1689, 1639, 1607, 1566. **HRMS** (MNa^+): calc. 629.1732, found: 629.1732.

Synthesis of 7e

1.47 g 1-adamantanecarbonyl chloride (7.01 mmol; 2.10 eq.) was added to a solution of **2a** at -70°C [prepared from 2.00 g (3.34 mmol; 1.00 eq.) **1** with 0.26 g (6.68 mmol; 2.00 eq.) potassium in 12ml THF]. Afterwards, the reaction mixture was brought to room temperature and stirred for another hour. The solution was added to 200 ml of saturated NH_4Cl solution and ice. After phase separation, three-fold washing of the aqueous phase with 100 ml of Et_2O, drying of the combined organic layers with Na_2SO_4 and evaporation of the solvents in vacuum, the product was purified via flash column chromatography (toluene/pentane 2:1) to result a yellow crystalline solid. Yield: 0.97 g (1,40 mmol; 42 %) of analytically pure **7e**.

7e: mp: 71°C. **Anal. Calc.** for $C_{42}H_{52}GeO_4$: C, 72,74; H, 7,56 % **Found**: C 72,67; H 7.30, %. ^{13}**C-NMR** (C_6D_6, TMS, ppm): 234.93, 234.08 (*C*=O), 143.57 (Mes-*C4*), 139.35 (Mes-*C1*), 133.49 (Mes-*C2*), 129.25 (Mes-*C3*), 53.48, 37.50, 36.69, 28.50 (Ad-*C*), 20.98, 19.84 (Mes-

CH_3). **^{1}H-NMR** (C_6D_6, TMS, ppm): 6.51 (s, 4H, Mes-H), 2.33 (s, 12H, Mes-C*H*$_3$), 2.00 (s, 6H, Mes-C*H*$_3$), 1.84 (s, 18H, Ad-C*H*$_2$ & -C*H*), 1.53 (s, 12H, Ad-C*H*$_2$). **UV-Vis:** λ [nm] (ε [L mol^{-1} cm^{-1}]) = 367 (939). **IR** (neat): ν(C=O) = 1648, 1607. **HRMS** (MNa^+): calc. 717.2986, found: 717.2999.

Synthesis of 7f

0.88 ml pivaloyl chloride (7.01 mmol; 2.10 eq.) was added to a solution of **2a** at -70°C [prepared from 2.00 g (3.34 mmol; 1.00 eq.) **1** with 0.26 g (6.68 mmol; 2.00 eq.) potassium in 12ml THF]. Afterwards, the reaction mixture was brought to room temperature and stirred for another hour. The solution was added to 200 ml of saturated NH_4Cl solution and ice. After phase separation, three-fold washing of the aqueous phase with 100 ml of Et_2O, drying of the combined organic layers with Na_2SO_4 and evaporation of the solvents in vacuum, the product was purified via flash column chromatography (toluene/pentane 2:1) to result a yellow crystalline solid. Yield: 0.79 g (1,36 mmol; 41 %) of analytically pure **7f**.

7f: mp: 105-108°C. **Anal. Calc.** for $C_{30}H_{40}GeO_4$: C, 67.07; H, 7,50 % **Found**: C, 67.23 H, 7.58%. **^{13}C-NMR** (C_6D_6, TMS, ppm): 235.35, 234.00 (*C*=O), 143.29 (Mes-*C4*), 139.35 (Mes-*C1*), 133.27 (Mes-*C2*), 129.25 (Mes-*C3*), 50.81 (-*C*-CH_3), 25.21 (-C-*C*H_3), 20.98, 19.78 (Mes-*C*H_3). **^{1}H-NMR** (C_6D_6, TMS, ppm): 6.49 (s, 4H, Mes-H), 2.27 (s, 12H, Mes-C*H*$_3$), 1.97 (s, 6H, Mes-CH3), 1.03 (s, 18H, -C-*CH*$_3$). **UV-Vis:** λ [nm] (ε [L mol^{-1} cm^{-1}]) = 363 (759), 273 (5450). **IR** (neat): ν(C=O) = 1642, 1628, 1607. **HRMS** (MNa^+): calc. 561.2043, found: 561.2016.

Synthesis of 7g

0.40 ml 1,4-dibromobutane (3.34 mmol; 1.00 eq.) was added to a solution of **2a** at -70°C [prepared from 2.00 g (3.34 mmol; 1.00 eq.) **1** with 0.26 g (6.68 mmol; 2.00 eq.) potassium in 12ml THF]. Afterwards, the reaction mixture was brought to room temperature and stirred for another hour. The solution was added to 200 ml of saturated NH_4Cl solution and ice. After phase separation, three-fold washing of the aqueous phase with 100 ml of Et_2O, drying of the combined organic layers with Na_2SO_4 and evaporation of the solvents in vacuum, the product was purified via flash column chromatography (toluene/pentane 2:1) to result a yellow crystalline solid. Yield: 0.51 g (1,21 mmol; 36 %) of analytically pure **7g**.

7g: mp: 71-72°C. **Anal. Calc.** for $C_{24}H_{30}GeO_2$: C, 68.13; H, 7,15 % **Found**: C, 68.34 H, 7.35 %. **^{13}C-NMR** (C_6D_6, TMS, ppm): 239.96 (*C*=O), 142.89 (Mes-*C4*), 138.78 (Mes-*C1*), 132.35 (Mes-*C2*), 129.13 (Mes-*C3*), 28.88 (Ge-CH_2-*C*-), 21.05, 19.21 (Mes-*C*H_3), 15.40 (Ge-*C*H_2-C-). **^{1}H-NMR** (C_6D_6, TMS, ppm): 6.52 (s, 4H, Mes-H), 2.12 (s, 12H, Mes-C*H*$_3$), 2.02 (s, 6H, Mes-C*H*$_3$), 1.52 (s, 4H, Ge-C*H*$_2$-CH_2-), 1.15 (s, 4H, -CH_2-C*H*$_2$-). **UV-Vis:** λ [nm] (ε [L mol^{-1} cm^{-1}]) = 363 (1191), 272 (7681). **IR** (neat): ν(C=O) = 1657, 1630, 1607. **HRMS** (MNa^+): calc. 447.1360, found: 447.1402.

DFT Calculations

The calculations of the optical absorption spectra are based on the crystal structure of **2b** including the two crown ethers and two potassium counter ions. Density functional theory was applied using the B3LYP[4] functional supplemented by Grimme's dispersion correction with Becke-Johnson damping D3BJ[5] (B3LYP-D3BJ), and the def2-SVP[6] basis set was used. For the simulation of the absorption spectrum, 30 vertical excitations were calculated by time-dependent DFT (TD-DFT) using the def2-TZVPP basis set.[7] The UV/Vis spectrum was simulated by a Lorentzian broadening with FWHH of 3000 cm^{-1} (the same value as the first peak in the experimental spectrum) using the orca_asa program.[8] The solvent THF was modelled by the conductor-like polarizable continuum model (CPCM).[9] The program ORCA4.2.1 was used for all simulations.

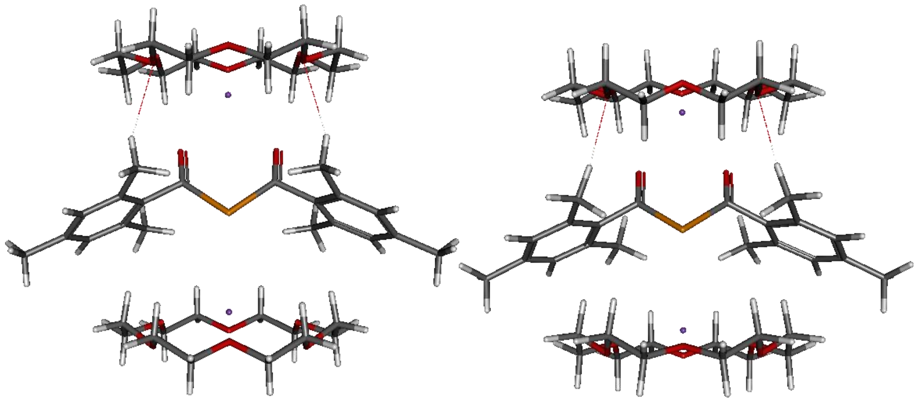

Figure S1. Crystal structure RF1873.pdb (left) and optimized DFT structure of **2b** (right). The violet dot represents the K^+ counter-ion.

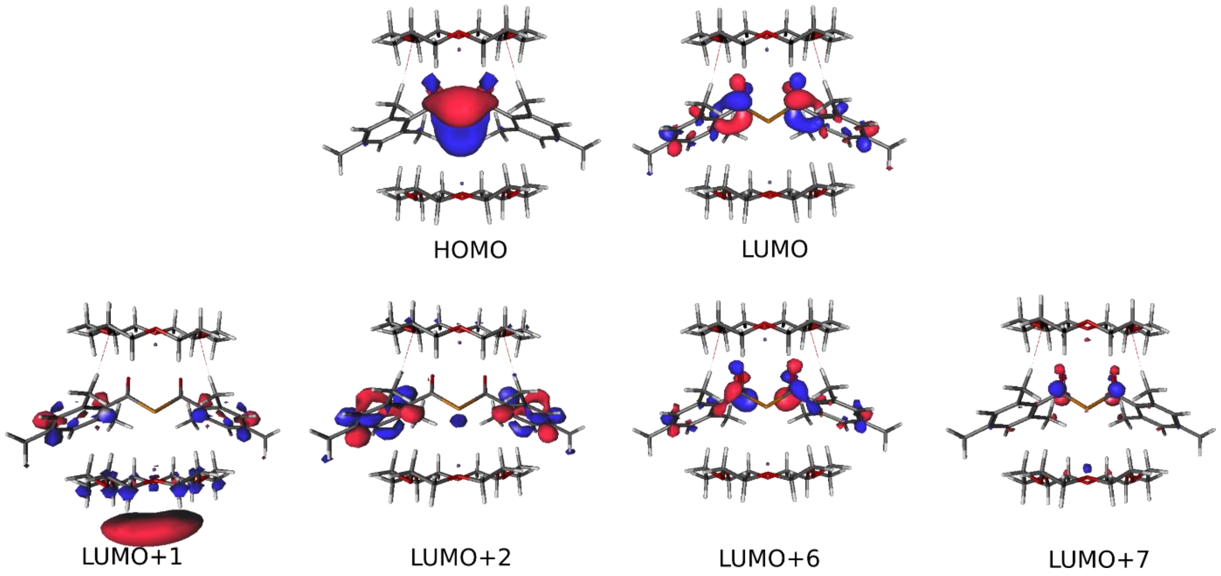

Figure S2. Relevant orbitals for the three most intense vertical excitations (S1, S9, S10). Contour values of 0.025 a.u. were chosen.

DFT geometry optimization retends the X-ray structure with a 10 degree shift of the mesityl groups relative to the K^+-Ge-K^+ axis (Figure S1), see Table S1 for a comparison of geometry data. The calculated absorption spectrum agrees well with the experimental one (see Figure 2 in the manuscript). The relevant transitions are listed in Table S2. The orbitals of the HOMO and LUMO are shown in Figure 2 in the manuscript, the other relevant orbitals for the intense bands are depicted in Figure S2.

Table S1. Relevant geometry parameters (distances d, angles α, dihedral angles γ) for compound **2b:** DFT optimized geometry (see Table S3 for Cartesian coordinates) compared to the crystal structure RF1893.pdb.

Distances /pm and angles /°	DFT	X-ray geometry
d(Ge-C1)	199.85	198.4(3)
d(C1=O)	126.10	125.2(4)
d(C1-C2)	152.51	154.6(4)
d(Ge-K1)	400.41	396.6(12)
d(Ge-K2)	343.69	333.6(11)
d(O-K1)	266.31	264.8(2)
d(O-K2)	564.36	578.6
α(C1-Ge-C1#)	97.93	103.64(19)
α(Ge-C1=O)	129.70	132.27
α(Ge-C1-Car)	116.78	113.63
α(C1=O…K1)	115.13	111.79
γ(Ge-C1=O…K1)	-40.94	-43.75
γ(O=C1-C2-C_7)	70.85	79.41

Table S2. The first five vertical excitations, the oscillator strength f and the orbital contributions as computed by TDDFT method B3LYP-D3BJ/def2-TZVPP/CPCM(THF). For the relevant excitations, the squared contributions c^2 of the LCAO coefficients are printed for c^2>0.15.

S1	λ / nm	578.3
	f	0.1516
	MO contribution (c^2)	H→L (0.82), H→L+6 (0.13)
S2	λ / nm	581.7
	f	0.0056
	MO contribution (c^2)	H→L+1 (0.36), H→L+2 (0.30), H→L+3 (0.32)
S3	λ / nm	578.4
	f	0.0004
	MO contribution (c^2)	H→L+3 (0.42), H→L+4 (0.42)
S4	λ / nm	570.1
	f	0.0043
	MO contribution (c^2)	H→L+5 (0.98)
S5	λ / nm	560.5
	f	0.0003
	MO contribution (c^2)	H→L+1 (0.51), H→L+2 (0.48)
S9	λ / nm	411.8
	f	0.0723
	MO contribution (c^2)	H→L+6 (0.60)

S10	λ / nm	404.0
	f	0.0406
	MO contribution (c^2)	H→L+7 (0.88)

Table S3. Cartesian coordinates (in .xyz format) of **2b**, optimized with DFT method.

131
optimized geometry of 2b, ORCA program, B3LYP-D3BJ/def2-SVP/CPCM(THF)
K 11.77436056288110 6.39040613393006 2.15365151259528
Ge 14.42825064514559 6.39447591433948 -0.84466938159694
O 13.91638247346952 7.89484658968385 1.66340648568199
C 14.30354404219631 7.90035931883492 0.46330021086732
O 9.45511241892884 6.38343408999614 0.14683519291027
C 14.87304029920020 9.24407367448405 0.02051157956014
O 11.64125016840311 8.89782208801965 3.82935865833581
C 14.64562934463102 11.40823046319476 -1.06468743240438
H 14.05829131989493 12.09244890379322 -1.68673317583915
O 10.51388195916383 8.79465098132597 1.20277542567465
C 14.12955957711951 10.13266457835713 -0.78683128529477
O 12.65264177679670 6.39739598605097 4.80969613449732
C 15.88825337540603 11.82979327852564 -0.57441589045272
C 16.61205149407451 10.93513678882538 0.22626467343095
H 17.58892387590610 11.23668587389789 0.61953477325101
C 16.12408431368021 9.65923558174354 0.53463509672956
C 12.78113196034766 9.73206882583785 -1.33401212851428
H 12.29671784806464 10.57398286623481 -1.85356802975312
H 12.12176420359809 9.37842040504268 -0.52758656775486
H 12.87768972591733 8.88997425423575 -2.03744289900364
C 16.45799534267012 13.17711747234915 -0.94487217279108
H 15.66389287304724 13.90039369774128 -1.18832426700601
H 17.11148714983881 13.10208120494571 -1.83373207678177
H 17.07037301439325 13.59777085265843 -0.13132606027584
C 16.93161672220701 8.72314682766888 1.39477151091791
H 17.06533446326021 7.75927166317620 0.87414295530830
H 16.39795006324114 8.48986415926217 2.32881344044516
H 17.91694243759552 9.14755974483683 1.63863285620563
C 12.62270358563056 7.58530248992047 5.57124071433208
H 13.44801143908374 7.59092738365774 6.31048328743406
H 11.66894723505545 7.66568012876473 6.13084854604252
C 12.78651692118724 8.77076224448157 4.65082646614175
H 12.92356640055389 9.68396871591846 5.26505419961155
H 13.68311631836780 8.63328457739749 4.01966438849257
C 10.55623915367777 9.96789808751272 1.98862928526046
H 9.63899803857277 10.05173820638410 2.60548957513382
H 10.60939508811263 10.86289334826362 1.33780625468781
C 9.46441946444468 8.76747793054304 0.26194483554614
H 9.48603485367897 9.67656893710450 -0.37075627864627
H 8.48169869105755 8.73553112747988 0.77367710013409
C 9.62980415841558 7.55635987131850 -0.62211044685271
H 8.88182570165636 7.60057882984407 -1.43885194125363
H 10.63874270655800 7.56147262293843 -1.07784384417032
C 11.77985261115183 9.93294015816054 2.86968968267925
H 12.67961486459544 9.74261783211011 2.25766185144353
H 11.88251548480375 10.91444104489976 3.37559361138424
O 11.64979464234107 3.89067923166791 3.83983467276205
O 10.51654620194650 3.97860868234161 1.21345779985235
C 12.63068238255390 5.21191622458991 5.57517883869815
H 13.45824450210906 5.21256002223092 6.31199822694087
H 11.67899218504127 5.12884375333168 6.13791126794546

C	12.79703779701189	4.02459521645440	4.65757123539902
H	12.93947439626329	3.11355377174459	5.27371762851640
H	13.69144997576575	4.16448482985964	4.02376689097721
C	10.56286227516668	2.81008029850108	2.00615276915095
H	9.64725779319714	2.72829966472437	2.62562722403430
H	10.61589775479776	1.91125570230301	1.36052734670984
C	9.46281603857545	3.99984487458231	0.27711745114499
H	9.48177360351543	3.08689463672444	-0.35013438600382
H	8.48248445311225	4.03459999429807	0.79317110447543
C	9.62430864055263	5.20537995572234	-0.61545594965450
H	8.87168946746564	5.15683114919330	-1.42767004290336
H	10.63038798705557	5.19627921007613	-1.07719094267144
C	11.78864182335001	2.85209564846509	2.88396095188081
H	12.68605734682991	3.04259879075980	2.26851588131765
H	11.89551234928734	1.87293822602503	3.39355119870095
K	17.46008504329120	6.39113475018246	-2.46342957908488
O	20.07876437688681	6.38729926892719	-1.05993418421187
O	18.78085293215147	3.99247879607751	-1.76874019194489
O	17.01178550835782	3.93452086100366	-4.03455076179274
O	15.89880223066507	6.39753819043886	-4.80806343610172
C	20.21247798239243	5.20509748824096	-0.29587342852739
H	19.44189800125995	5.17300810133796	0.49610657916925
H	21.20479933255483	5.16861425387112	0.19505899504074
C	20.07048442780420	4.00399271330644	-1.19734943427203
H	20.84427718473563	4.03283979101504	-1.99033644439693
H	20.23808573172998	3.08866464437212	-0.59651987918816
C	18.51957750824569	2.83630607598647	-2.53898224885168
H	18.62378695778108	1.93034307047813	-1.91378077717786
H	19.24229146971937	2.76030731617289	-3.37591873686260
C	17.11307493165280	2.89734191012589	-3.07415306535553
H	16.86017308987044	1.92147056973937	-3.53254865839634
H	16.40725188966195	3.07496496783240	-2.24722140118289
C	15.70511591847655	4.04982141979725	-4.56444688876069
H	14.97758238937747	4.22138170056277	-3.74747440346369
H	15.41800391425324	3.11990582659281	-5.09423927026842
C	15.65705649561723	5.20982955278001	-5.52801815596036
H	16.40924739932609	5.07629081094273	-6.33149157862634
H	14.65660243130701	5.23808703080482	-6.00210433461893
O	18.78094901592969	8.78701633477332	-1.75088806787682
O	17.01805769255754	8.85607453952392	-4.02152697761795
C	20.20894822400619	7.56246927837894	-0.28446893404930
H	19.43593899386031	7.58655042836198	0.50552059929014
H	21.19975107274267	7.59536025574073	0.20973659831355
C	20.06866266774872	8.77152443602237	-1.17549341910172
H	20.84501948287149	8.74991989605994	-1.96625395039941
H	20.23391817357289	9.68175786912789	-0.56633155939357
C	18.52459614851680	9.94616443587142	-2.51845213660336
H	18.62895733224549	10.85013396499188	-1.89039986248088
H	19.25008608837683	10.02282609594957	-3.35296624409143
C	17.11970813270151	9.89056589371535	-3.05818431897113
H	16.87139226595858	10.86842036955410	-3.51476433276332
H	16.41071052979186	9.71274125803087	-2.23407708906889
C	15.71170419596411	8.74486801434115	-4.55284607587115
H	14.98369126654278	8.57076334229330	-3.73680389981526
H	15.42593991147699	9.67772192888696	-5.07823657319246
C	15.66193410307183	7.58975659298600	-5.52212487560479
H	16.41580883795912	7.72508489640027	-6.32369318425941
H	14.66218034168334	7.56687439766237	-5.99797828314749
O	13.91642383076209	4.88699620468690	1.65935946600272
C	14.30384398408782	4.88537809228155	0.45944182327303
C	14.87563447476673	3.54385149122614	0.01351705154841

C	14.64918901564700	1.37952573001780	-1.07146130590838
H	14.06161815028843	0.69452112366677	-1.69238464738097
C	14.13208168941494	2.65450620545405	-0.79271106806966
C	15.89299327017030	0.95940200073243	-0.58269439143625
C	16.61689087870058	1.85481426311477	0.21711740624458
H	17.59437381270516	1.55408164729109	0.60951064249222
C	16.12788058642824	3.13012155504973	0.52608798903203
C	12.78178398579644	3.05378438030571	-1.33628977408594
H	12.29999272647906	2.21377737518997	-1.86132315098397
H	12.12183338263056	3.39875456424618	-0.52646830079256
H	12.87477650428271	3.90164346837132	-2.03334106618616
C	16.46337031809857	-0.38785197620969	-0.95224771040052
H	15.67009985193579	-1.10818199585695	-1.20662726894417
H	17.12687539114717	-0.31177797040532	-1.83356180894319
H	17.06604265317335	-0.81296031952673	-0.13372489732132
C	16.93417738108517	4.06668050395096	1.38691324090621
H	17.07379824127830	5.02849259604563	0.86398433207883
H	16.39558749401682	4.30524195857606	2.31668656657041
H	17.91671996878664	3.64001812324252	1.63798515022829

Mechanistic Investigations

Cw-EPR measurements were performed with a Bruker EMX X-band EPR spectrometer (100 kHz field modulation) equipped with a variable-temperature unit (Eurotherm B-VT 2000). Typical conditions for the acquisition of the EPR spectra were 2 mW microwave power and 0.1 mT field modulation. The public domain program WinSim (in the versions 0.96 and 0.98)[10] was used to analyze and simulate the spectra.

A custom-made three-compartment EPR sample tube connected to a high-vacuum line was used for the preparation of a sample of compound **6**. A potassium metal mirror was sublimated to the wall of the tube, and the investigated compound was dissolved in freshly condensed THF (ca. 0.4 mL; stored over Na/K alloy). Afterward, the sample was degassed by three freeze−pump−thaw cycles and sealed under high vacuum. The reduction was performed by bringing the THF solution of **6** in contact with the K metal mirror in the evacuated sample tube.

UV−Vis spectra were acquired on a UV−Vis spectrometer equipped with optical fibers and a 1024-pixel diode-array detector (J&M Analytik AG, Essingen, Germany). The spectra were measured through the EPR capillary of the sample tube, which was fixed in a 1x1 cm quartz cuvette using 3D printed holders. The cuvette was filled with deionized water to minimize reflections. As a reference a similar cell filled with THF was used.

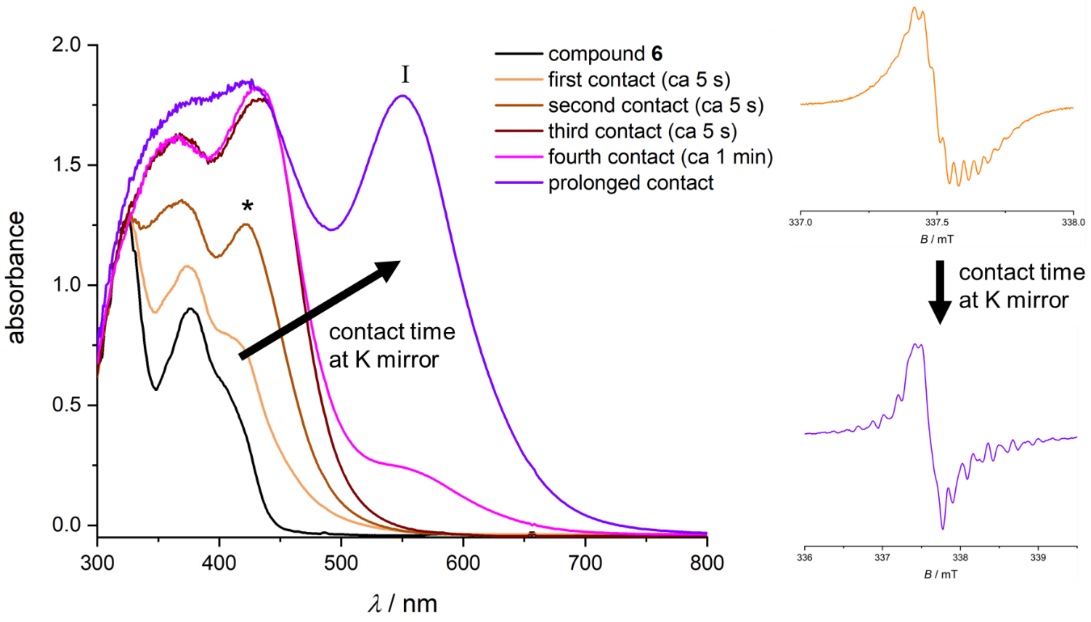

Figure S2. Left: UV-Vis spectra of **6** and reduction products in ultra-dry THF (inert conditions) in an EPR tube. Right: EPR spectra at the beginning (first contact; g = 2.0052 ± 0.0005) at the end of the experiment (prolonged contact; g = 2.0047 ± 0.0005) at T = 204 K.

Before performing the reductions, a UV-Vis spectrum of **6** in ultra-dry THF, which was prepared in the special three-compartment EPR cell, was acquired (black curve in Figure S2). Afterwards the solution was repeatedly brought in close contact with the potassium mirror. After each contact, UV-Vis and EPR spectra were acquired (see additional curves in Figure S2 and EPR spectra on the right side of the graph).

The UV-Vis spectra clearly show that at first a new peak is formed at about 425 nm (marked with an asterisk) indicating the formation of the germenolate **1**.[11] At the same time, the EPR spectrum depicted in the right left corner is obtained. Further contact at the K metal mirror leads

to the formation of a new peak at about 550 nm, which is attributed to the dianion **2a** (marked with I). During the experiment, the colour of the solution changed from yellowish (i.e. the colour of **6**) over orange to reddish brown (germenolate **1**) to violet (dianion **2a**). The EPR spectrum of the solution also substantially changed upon prolonged contact.

In both cases, the EPR spectra are a superposition of at least two different species making an interpretation very tedious. A part of the first spectrum could be simulated with the parameters shown in Table S2. The magnitude of the hyperfine coupling constants are comparable to the ones obtained for the radical anion of mesitoyl-substituted phosphorus-based photoinitiator BAPO, where the spin density is delocalized over two the mesitoyl groups (see Table for hyperfine coupling constants).[12]

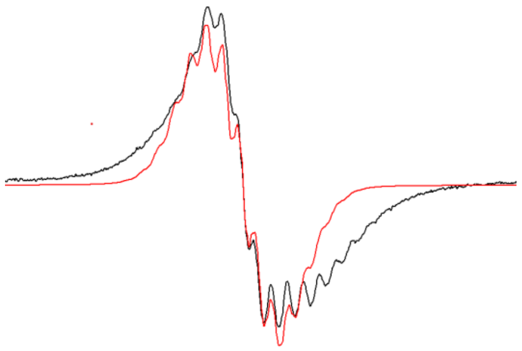

Figure S3. EPR spectrum (black curve) after the first contact of a solution of **6** in THF with K metal mirror and simulation in red.

Table S2. Hyperfine coupling constants for the EPR spectrum shown in Figure S3 and comparison to the BAPO radical anion.

	$\lvert a^H{}_p \rvert$ / mT	$\lvert a^H{}_o \rvert$ / mT	$\lvert a^H{}_m \rvert$ / mT
**species 1**	0.044 (6 H)	0.0315 (12 H)	0.009 (4 H)
BAPO radical anion[12]	0.060 (6 H)	0.039 (12 H)	0.012 (4 H)

Based on these observations, the EPR spectrum might be assigned to a radical anion in which the spin is evenly distributed among two mesitoyl units. This radical might be directly derived from the tetramesitoylgermane or might be the radical anion of the mesitoyl-substituted derivative of benzil (mesityl, $((MesC{=}O)_2)$), which is expected to be formed as by-product of the germa-acyloin condensation.

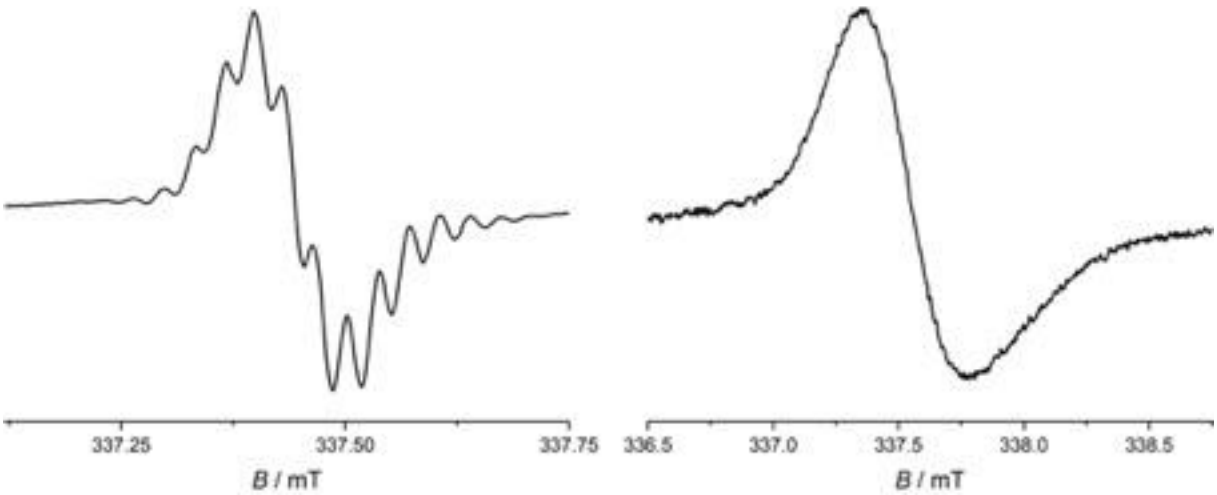

Figure S4. Left: EPR spectrum of a reaction mixture (**6** + potassium) in benzene + [18]-crown-6 (T = 290 K; g = 2.0053 ± 0.0005). Right: EPR spectrum of reaction mixture (**1** + potassium) in THF + [18]-crown-6 (T = 200 K; g = 2.0050 ± 0.0005).

In Figure S4 the rather well resolved EPR spectrum of the reaction mixture containing **1** and potassium as reducing agent in presence of [18]-crown-6 is shown. Quite interestingly, an almost identical spectrum was also described by us in a previous publication[11], where we tentatively attributed it to a follow-up product of **1** and the *tert*-butoxy radical derived from KO*t*Bu. Since no KOtBu was added to reaction mixture, the radical must be derived from tetramesitoylgermane.

CIDNP Experiments

CIDNP (chemically induced dynamic nuclear polarization) NMR experiments were carried out on a 200 MHz Bruker AVANCE DPX spectrometer equipped by a custom-made CIDNP probe head. A Quantel Nd-YAG Brilliant B laser (355 nm, ~50 mJ per pulse, pulse length 8–10 ns) operating at 20 Hz was employed as the light source. The pulse sequence of the experiment consists of a series of 180° radio-frequency (RF) pulses to suppress the NMR signals of the parent compounds, the laser flash, the 90° RF detection pulse and the acquisition of the free induction decay (FID). "Dummy" CIDNP spectra employing the same pulse sequence but without the laser pulse were always measured. Samples were prepared in toluene-d_8 and deoxygenated by bubbling with nitrogen before the experiment. Chemical shifts (δ) are reported in ppm relative to tetramethylsilane (TMS) using the residual methyl signal of deuterated acetonitrile as an internal reference (δ_H = 1.94 ppm). If necessary, line broadening (1 Hz, exponential) was applied to the spectra.

Results:

Investigated compounds

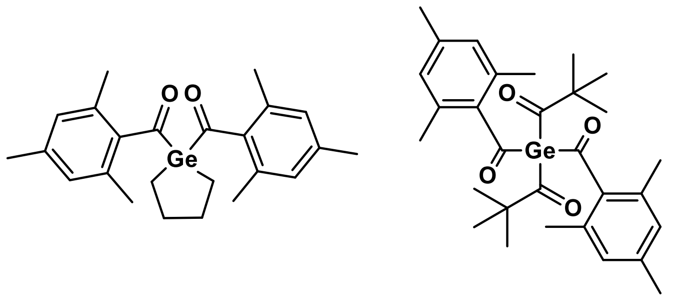

7g 7e

Figure S5. Chemical structures and abbreviations of the investigated compounds

In the Figure S6, ^{1}H NMR spectrum together with CIDNP spectrum of **7g** in the presence of butyl acrylate is shown in deuterated acetonitrile. The signal of mesitaldehyde (**MH**, δ = 10.54 ppm) is visible. Signals enclosed in the green rectangle can be assigned to the α-H proton of the radical reaction product **7g** with coupling constants ^2J = 11.1 Hz and ^3J = 6.0 Hz. Signal corresponds to well resolved doublet of doublets. Situation is slightly different in toluene-d_8 as a solvent (Figure S7). In this case the second order effects influence the coupling of α-H proton (marked in green) shifting its apparent coupling pattern towards that of a triplet resulting in a coupling constant of ^3J = 9.1 Hz and ^3J = 6.0 Hz. The signal of **MH** is visible at δ = 10.37 ppm. In both solvent, toluene-d_8 and acetonitrile-d_3, signals corresponding to the β-H protons of **7g** product, were not assigned though to the substantial overlap with other signals in aliphatic region.

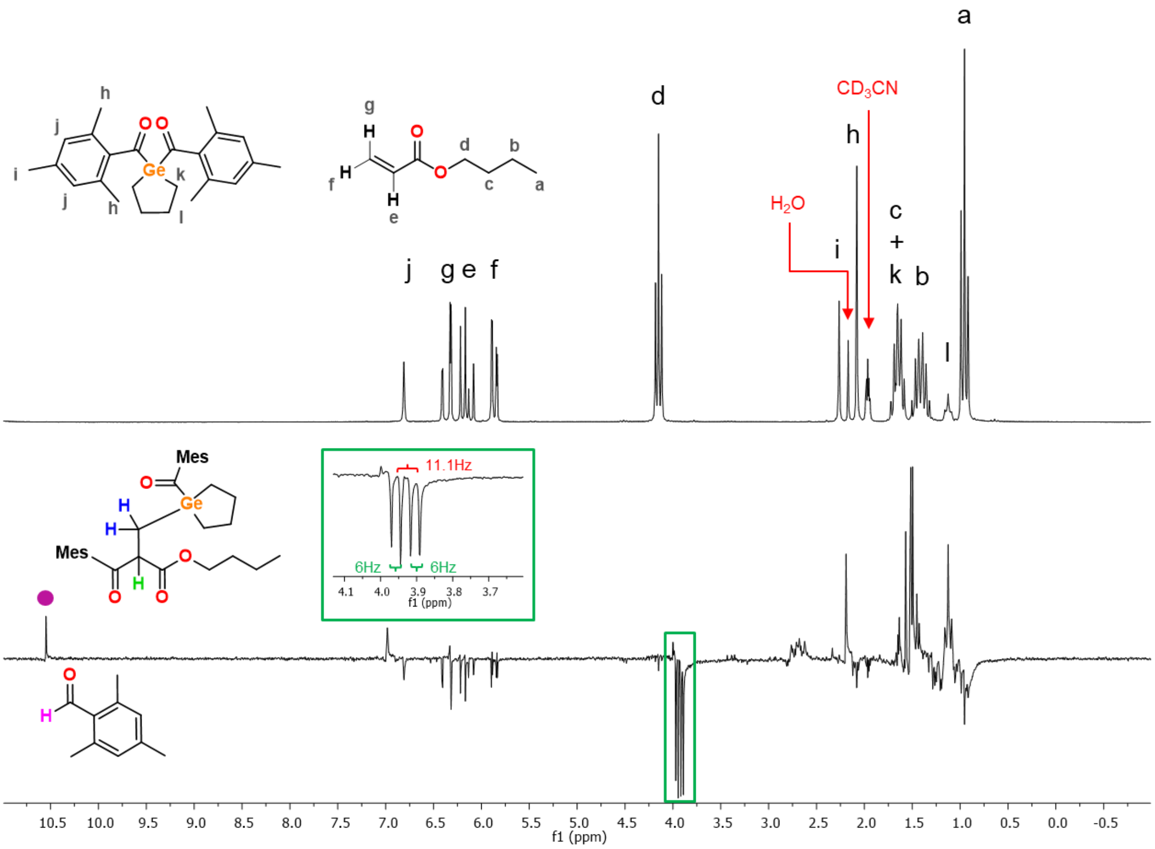

Figure S6. ^{1}H NMR and CIDNP spectra (excitation at λ = 355 nm, ca. 50 mJ per pulse) of **7g** in the presence of butyl acrylate (10 mM **7g**, 50 mM BA in acetonitrile-d_3).

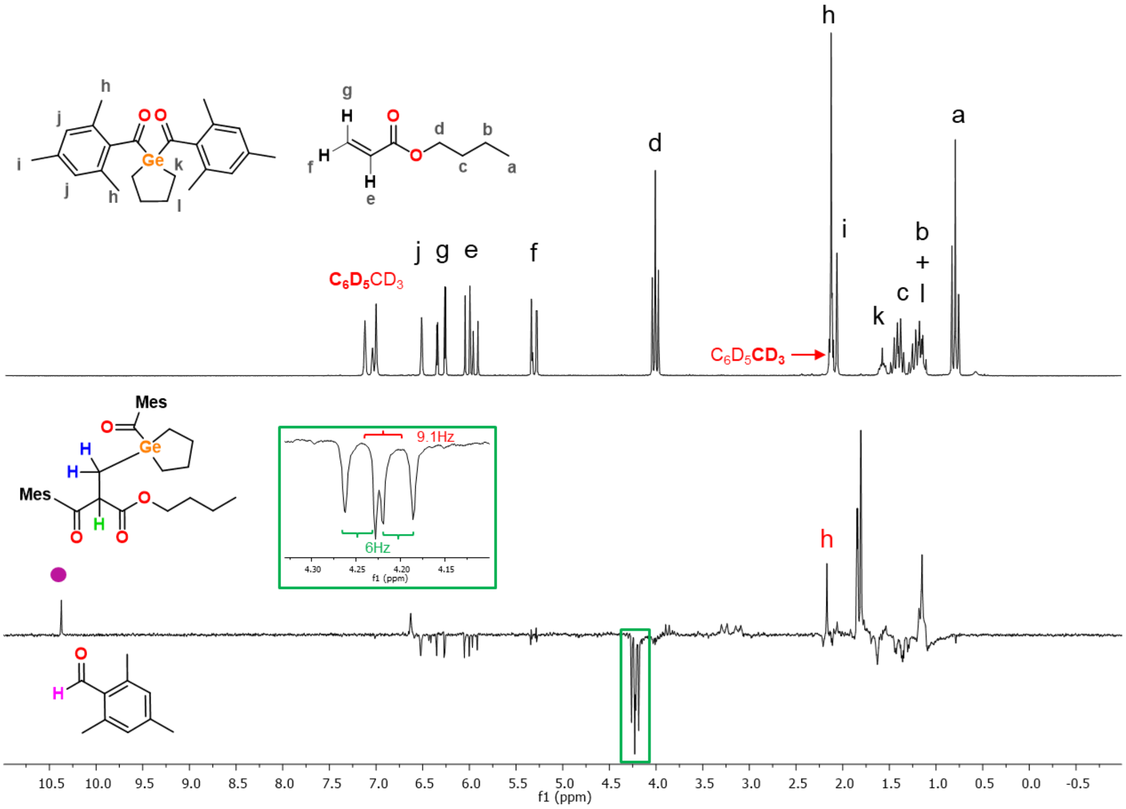

Figure S7. ^{1}H NMR and CIDNP spectra (excitation at λ = 355 nm, ca. 50 mJ per pulse) of **7g** in the presence of butyl acrylate (10 mM **7g**, 50 mM BA in toluene-d_8).

In the Figure S8. and S9. ^{1}H NMR spectrum and CIDNP spectrum of **7e** in the presence of butyl acrylate is shown using acetonitrile-d$_3$ and toluene-d$_8$ as solvents respectively. In both cases signal corresponding to **MH** is visible with shifts of δ = 10.53 ppm in case of acetonitrile-d$_3$ and δ = 10.37 ppm in case of toluene-d$_8$. Coupling of the α-H proton (marked in green, with the corresponding CIDNP signals in green rectangle) is presented in both solvents. Similar situation as in the case of the **7g** derivative is observed. In acetonitrile-d$_3$ as solvent doublet of doublets is observed with coupling constants ^2J = 16.2 Hz and ^2J = 6.0 Hz. Whereas in toluene-d$_8$ corresponding signals shift towards the apparent triplet shape though the second order effects with coupling constant of ^3J = 7 Hz. Once again signals corresponding to the β-H protons of **7e** product, could not be unambiguously assigned.

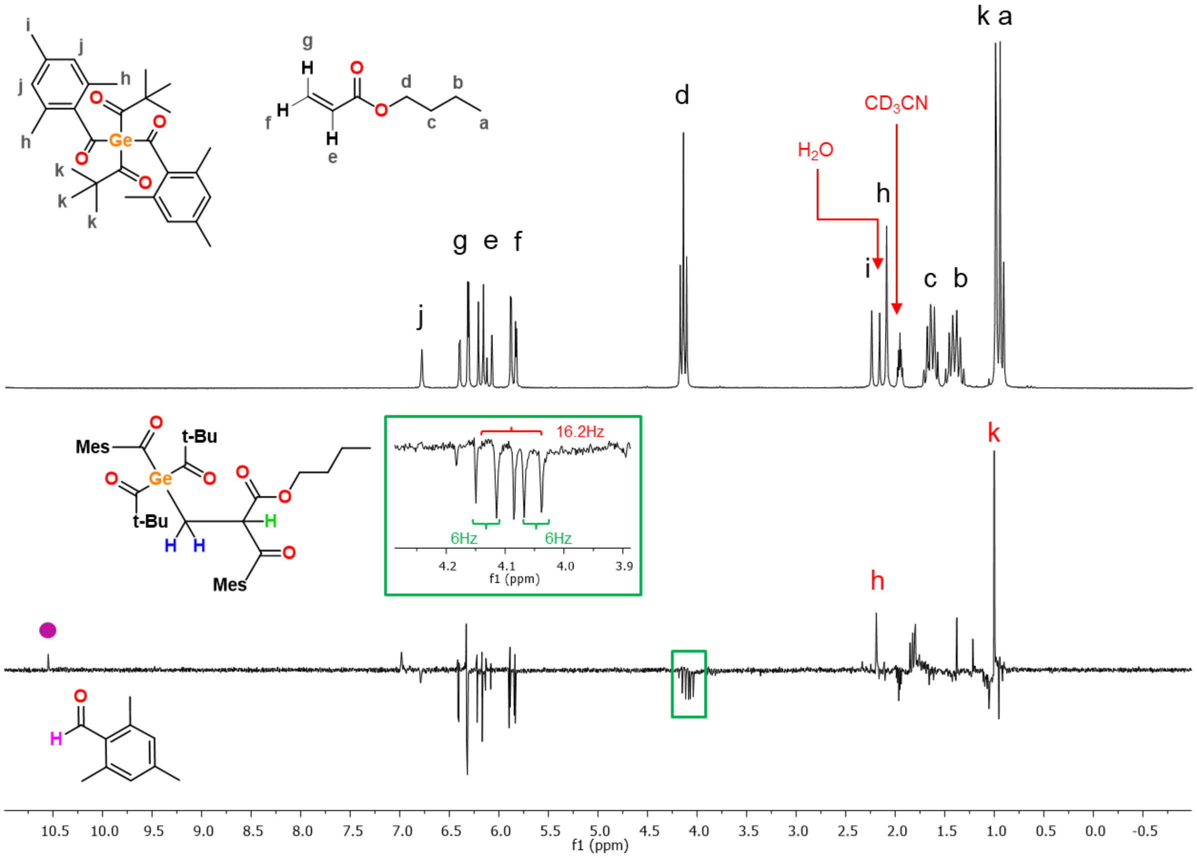

Figure S8. ^{1}H NMR and CIDNP spectra (excitation at λ = 355 nm, ca. 50 mJ per pulse) of **7e** in the presence of butyl acrylate (10 mM **7e**, 50 mM BA in acetonitrile-d$_3$).

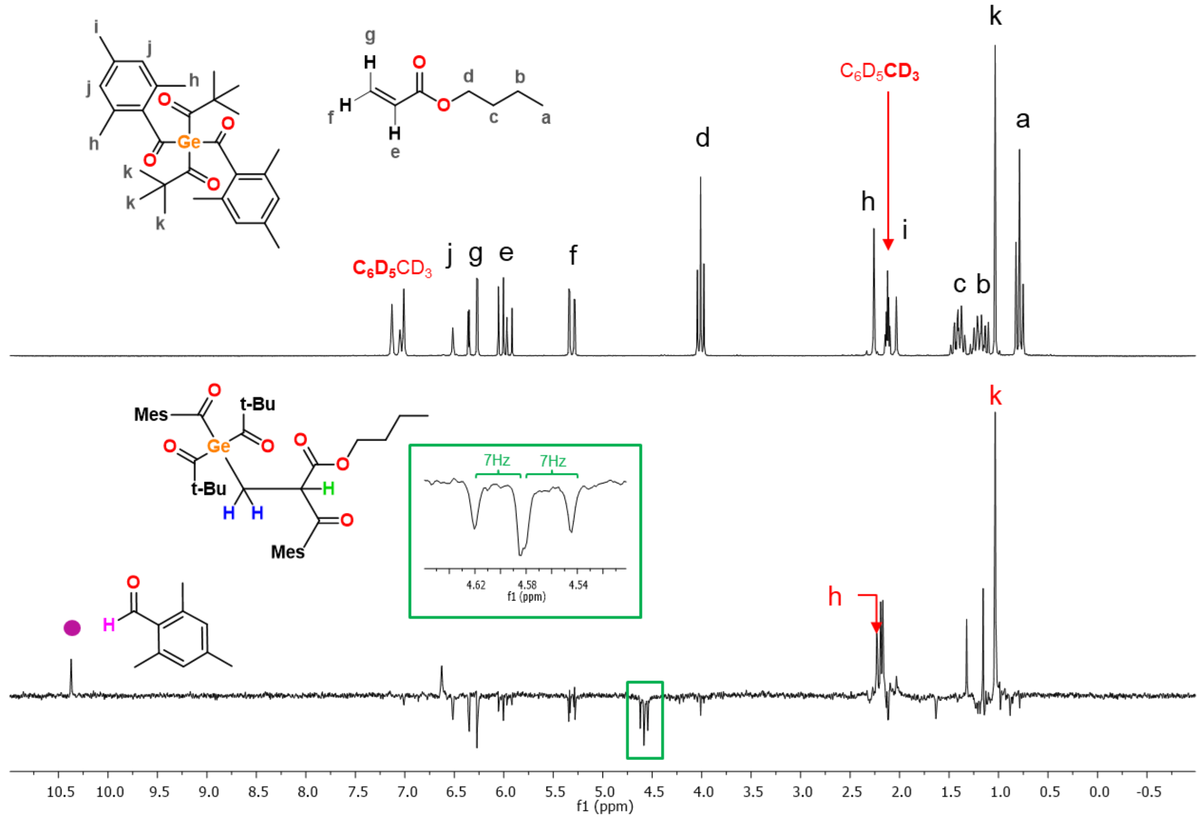

Figure S9. ^{1}H NMR and CIDNP spectra (excitation at λ = 355 nm, ca. 50 mJ per pulse) of **7e** in the presence of butyl acrylate (10 mM **7e**, 50 mM BA in toluene-d_8).

MAS NMR Experiments

NMR spectra under magic angle spinning (MAS) conditions were recorded with a 500 MHz Avance spectrometer (Bruker, 11.7 T). The experiments were performed at Larmor frequencies of 500 MHz and 202.4 MHz for ^{1}H and ^{31}P, respectively. All samples were filled in zirconia cylinders with 2.5 mm diameter inside a glove box under argon atmosphere.

For the ^{1}H measurements the P1 pulse length was set to 0.8 µs and the pulse power to 20 Watt. With a recycle delay of 10 s we accumulated 64 scans for the final spectrum. The experiments were performed at 31°C with a spinning speed of 30 kHz. The ^{1}H spectra were referenced to the signal of adamanthane. The ^{1}H spectra show an overlap of the different atoms; therefore the integrals of the respective signals show some uncertainty. Especially for compound **2b** the partly coordinated [18]-crown-6 molecule hinders the exact determination of the included H-atoms.

To increase the sensitivity for ^{13}C spectra and eliminate broadening from chemical shift anisotropy and dipolar coupling,[13] additionally to spinning the samples at 25 kHz in the magic angle (54.74° with respect to the main magnetic field), we performed polarization transfer experiments at 30°C. The contact time was set to 7 ms and we accumulated 8192 scans for the spectra with a recycle delay of 3 s. The ^{13}C spectra were referenced to the signal of adamanthane (upfield signal at 38.48 ppm from trimethylsilane). All C-atoms of compound **2a** and **2b** could be identified via MAS NMR. For the carbonyl-C the weak dipolar coupling to ^{1}H-nuclei and thereby hindered polarization transfer results in a small signal intensity.

NMR-Spectroscopy

Figure S10: ^{1}H- and ^{13}C-NMR spectra of $K_2Ge(C(O)Mes)_2$ (**2a**) (THF-d_8 solution, vs ext. TMS, ppm)

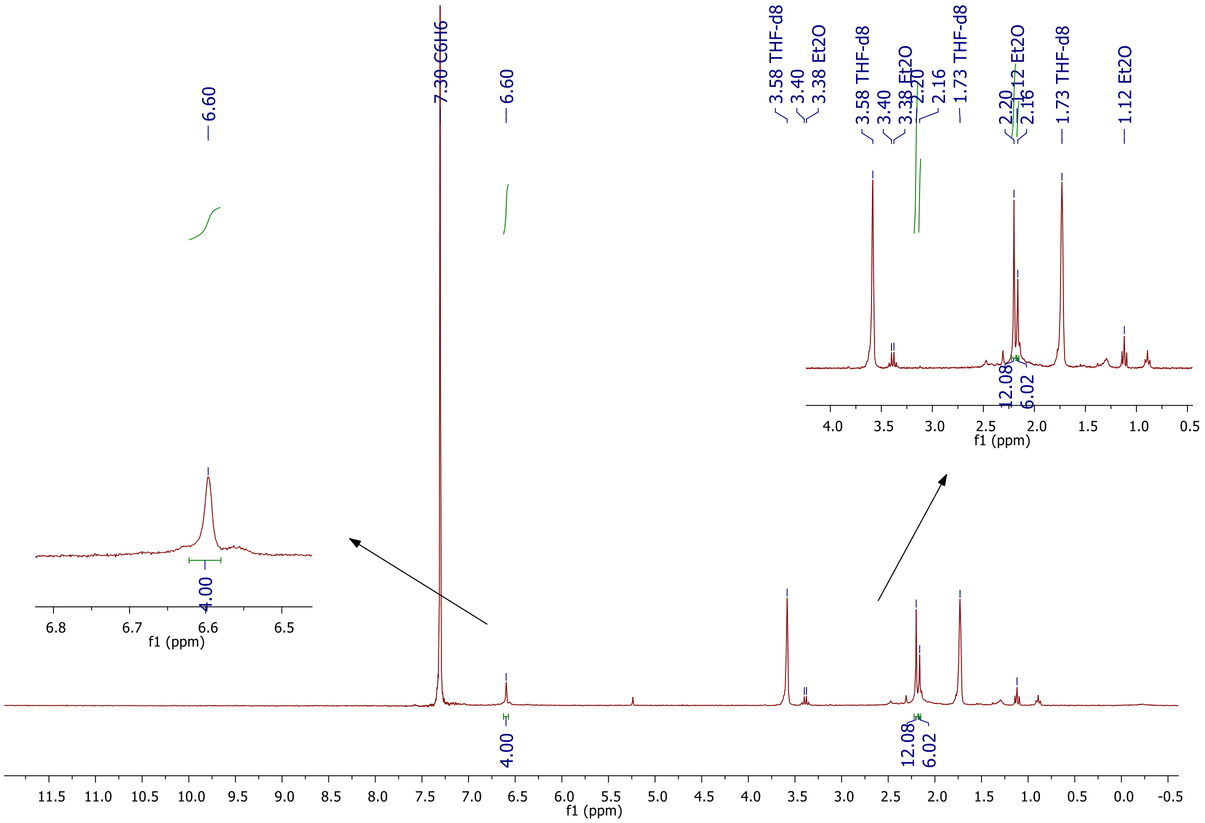

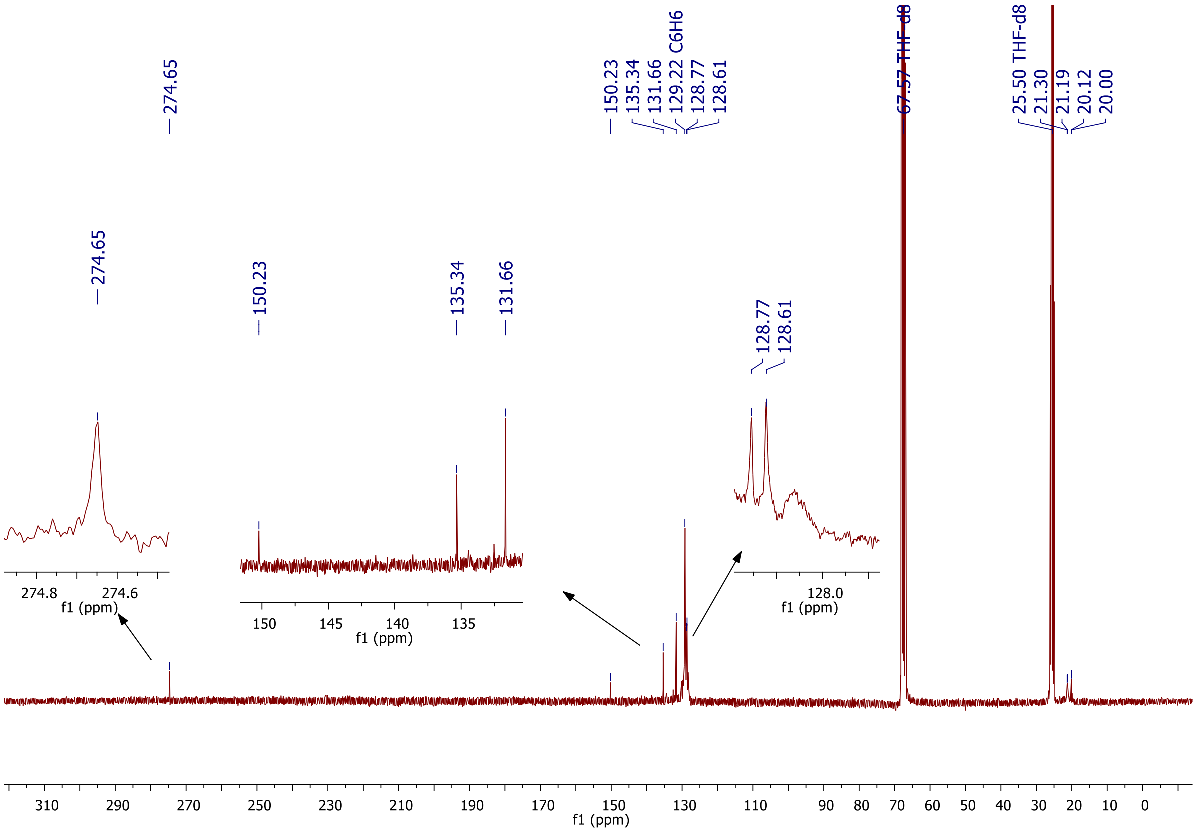

Figure S11: MAS ^{1}H- and ^{13}C-NMR spectra of $K_2Ge(C(O)Mes)_2$ (**2a**)

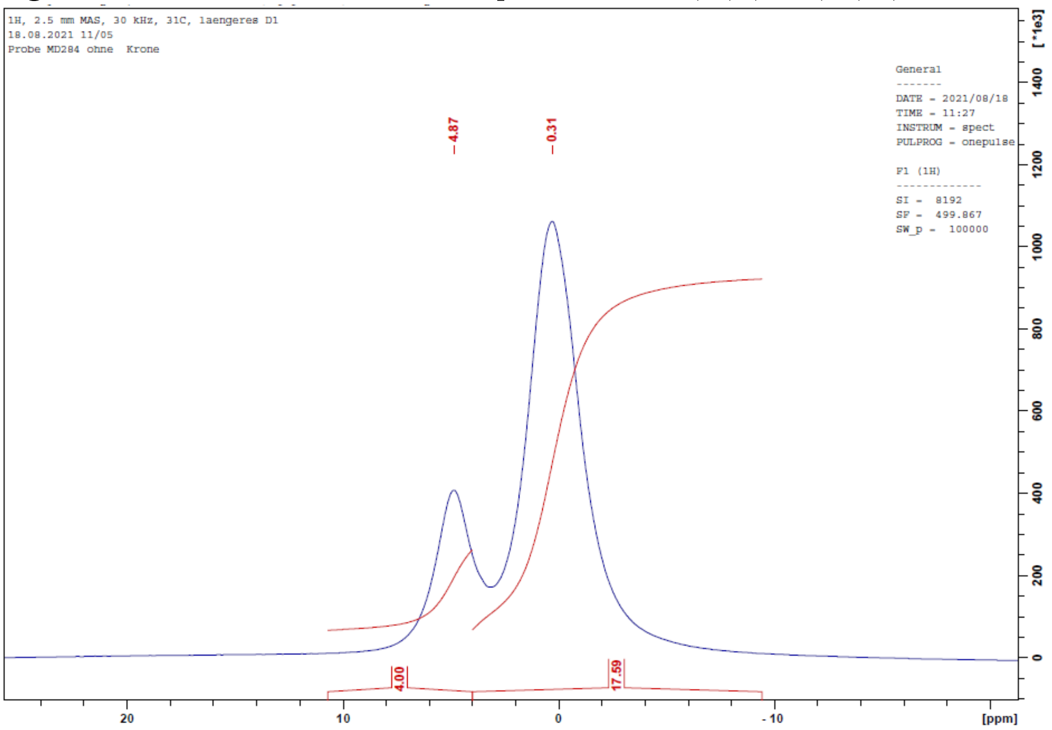

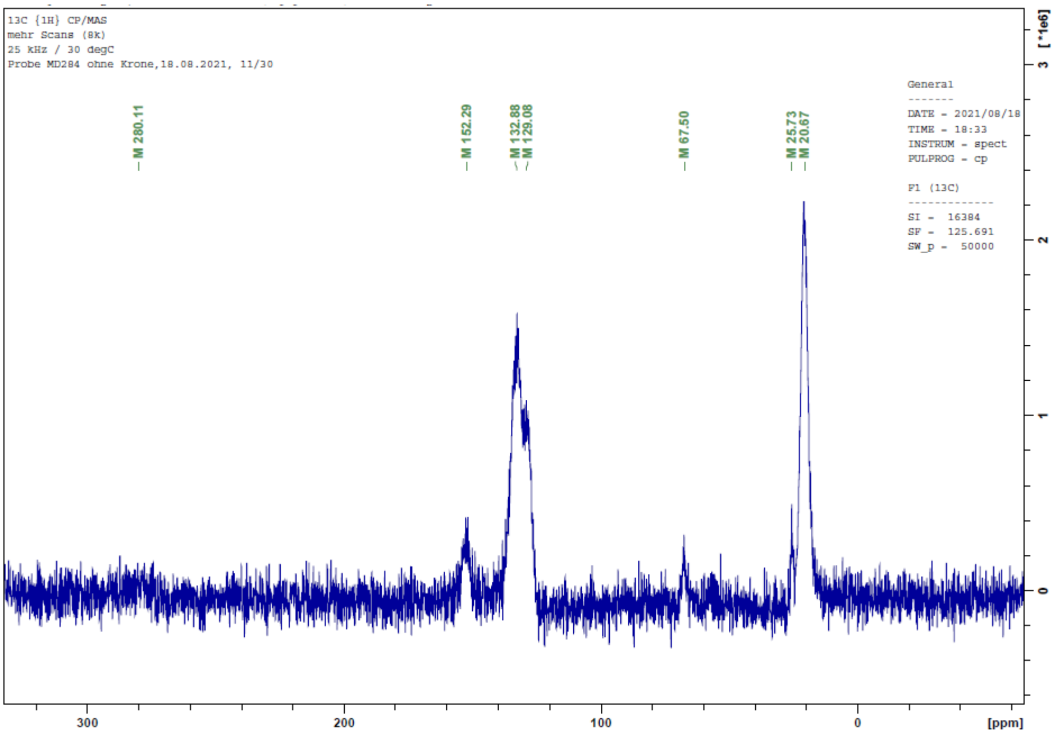

Figure S12: ^{1}H- and ^{13}C-NMR spectra of $K_2Ge(C(O)Mes)_2$*2 18-crown-6 (**2b**) (THF-d_8 solution, vs ext. TMS, ppm) (In the 13C-NMR spectrum of 2b we could identify **2a** as side product, which is marked with a *)

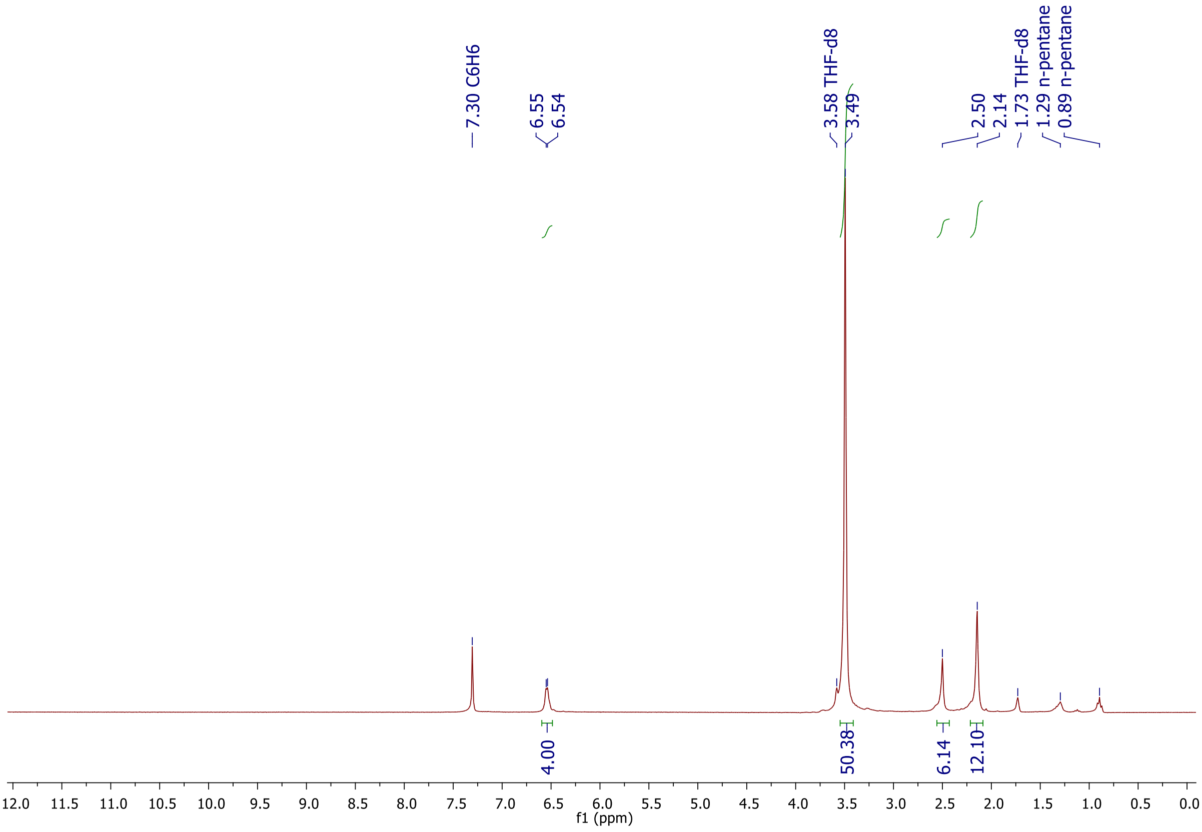

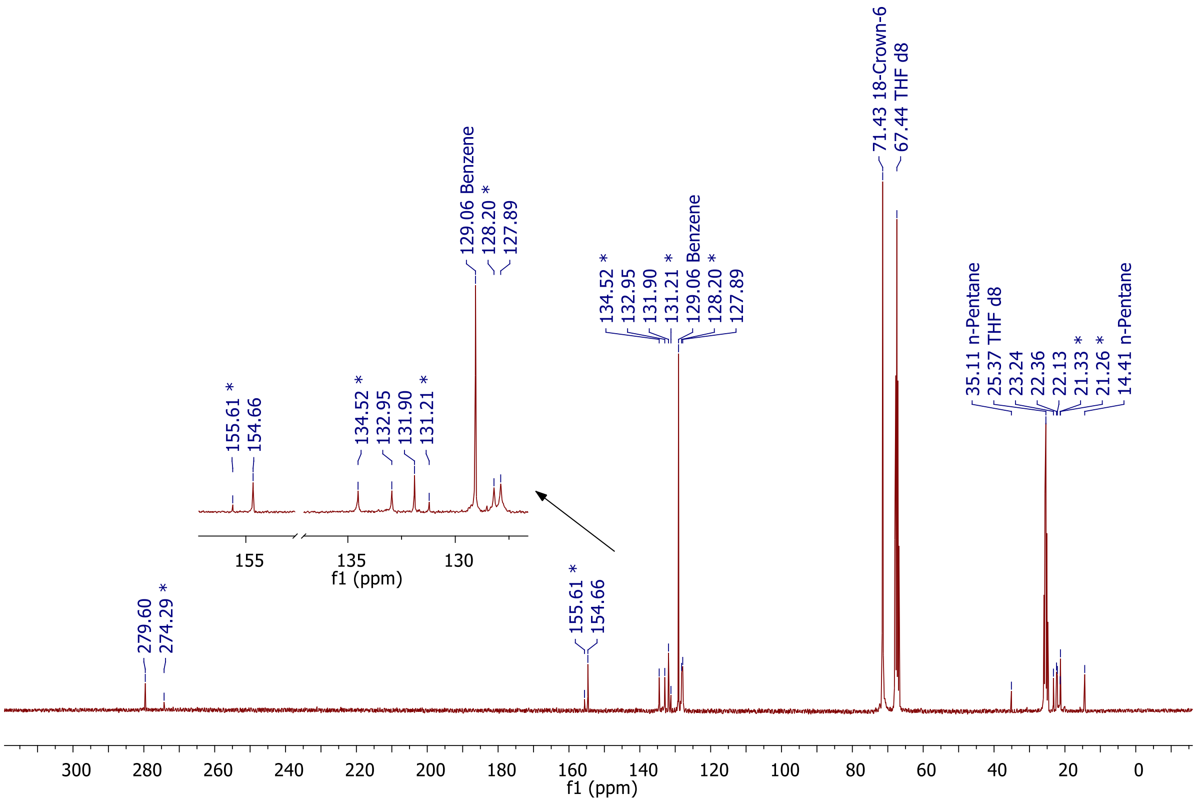

Figure S13: MAS ^{1}H- and ^{13}C-NMR spectra of $K_2Ge(C(O)Mes)_2$*2 18-crown-6 (**2b**)

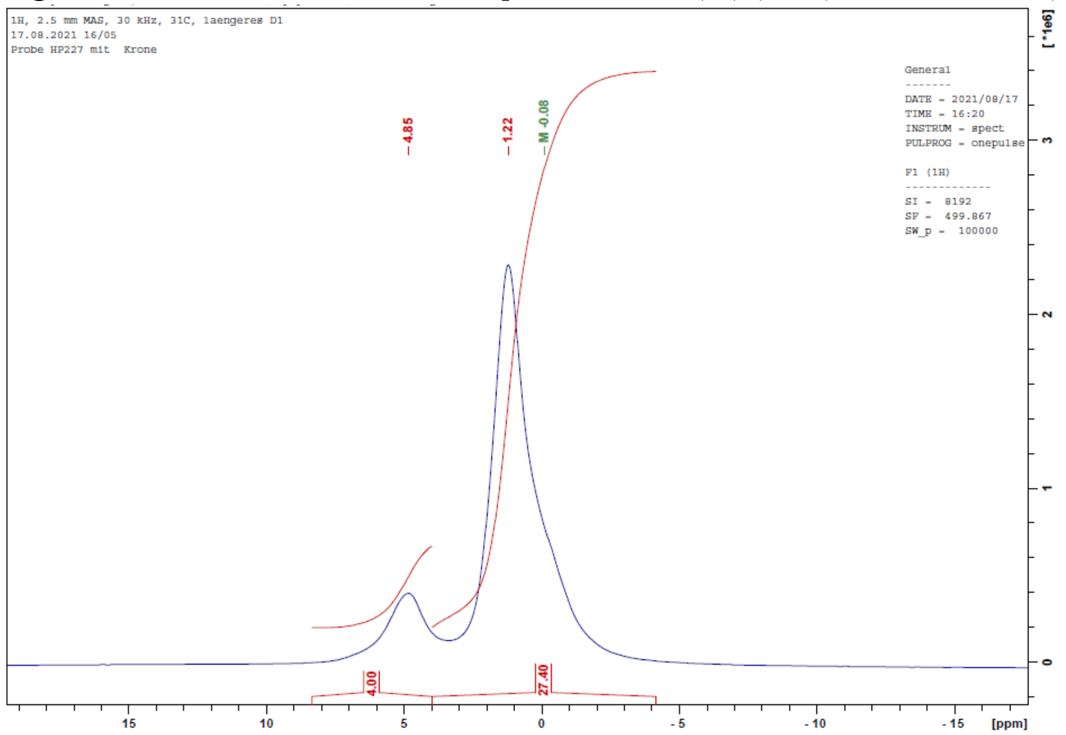

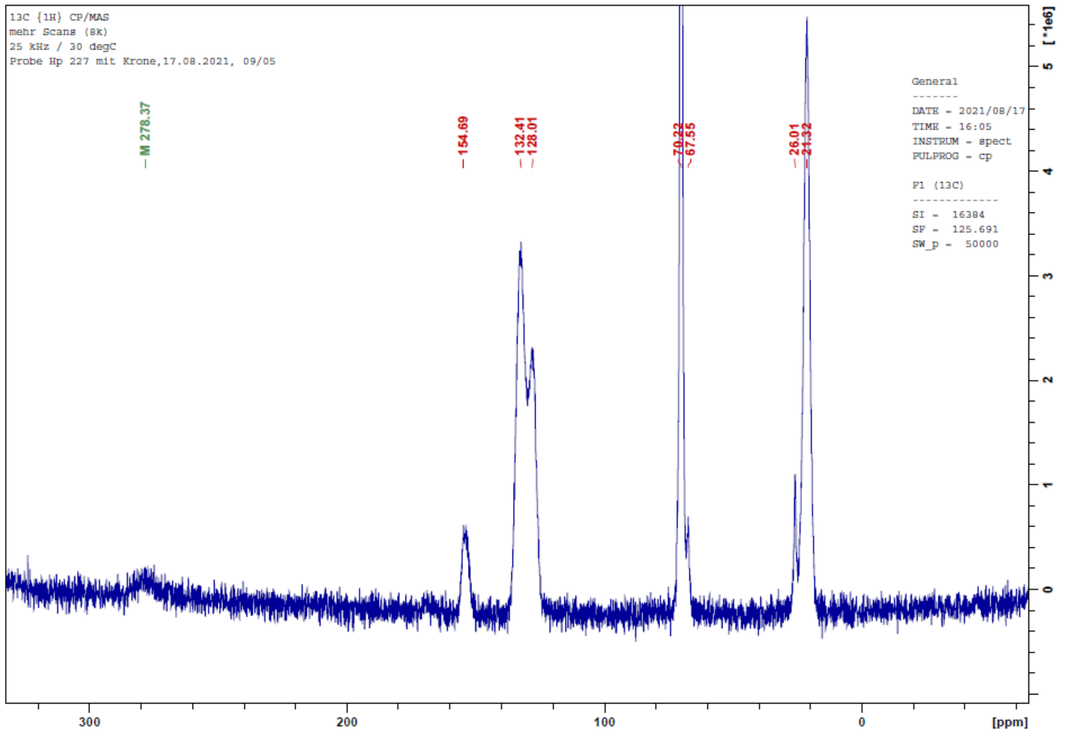

Figure S14: ^{1}H- and ^{13}C-NMR spectra of $(Me)_2Ge(C(O)Mes)_2$ (**7a**) ($CDCl_3$ solution, vs ext. TMS, ppm)

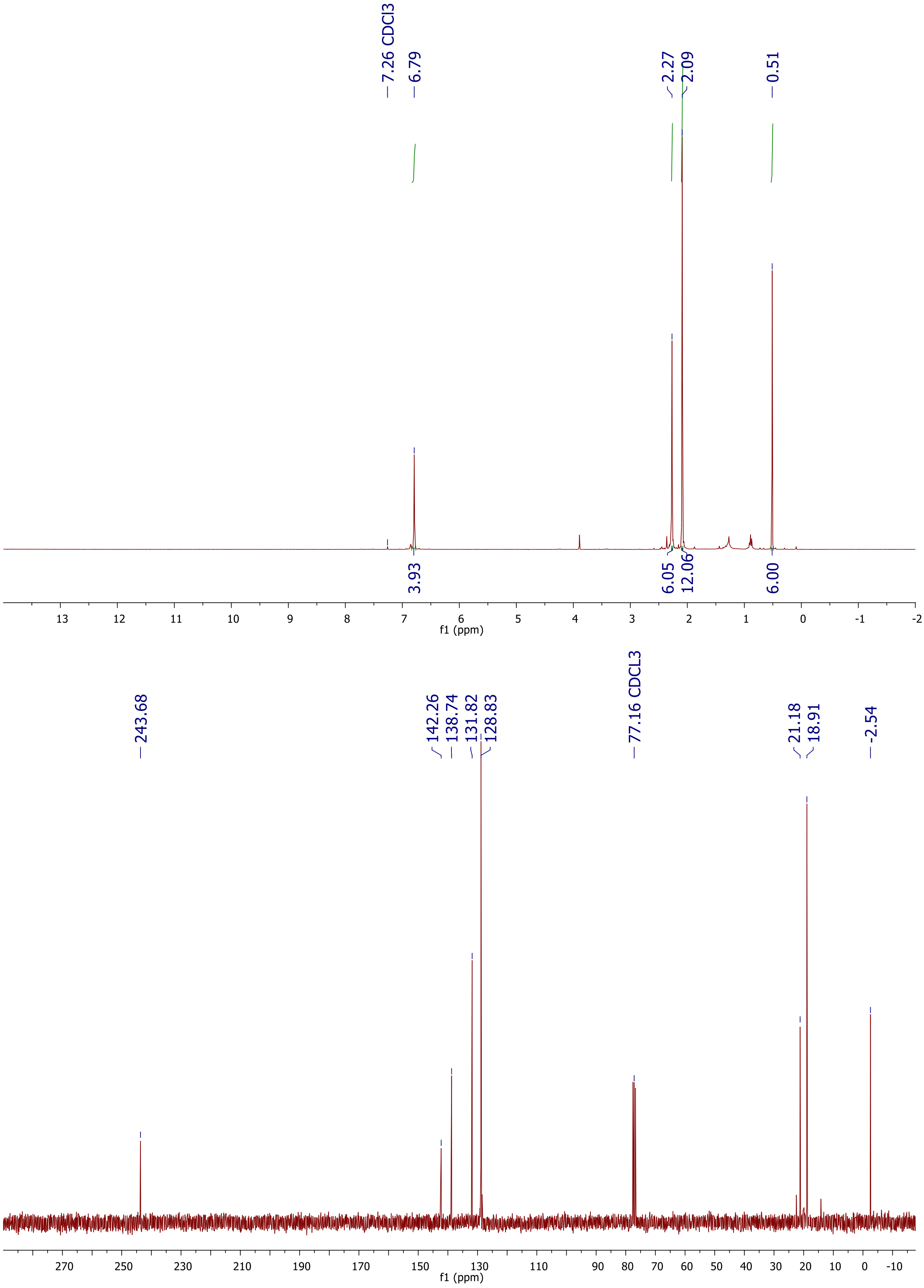

Figure S15: ^{1}H- and ^{13}C-NMR spectra of $(Et)_2Ge(C(O)Mes)_2$ (**7b**) ($CDCl_3$ solution, vs ext. TMS, ppm)

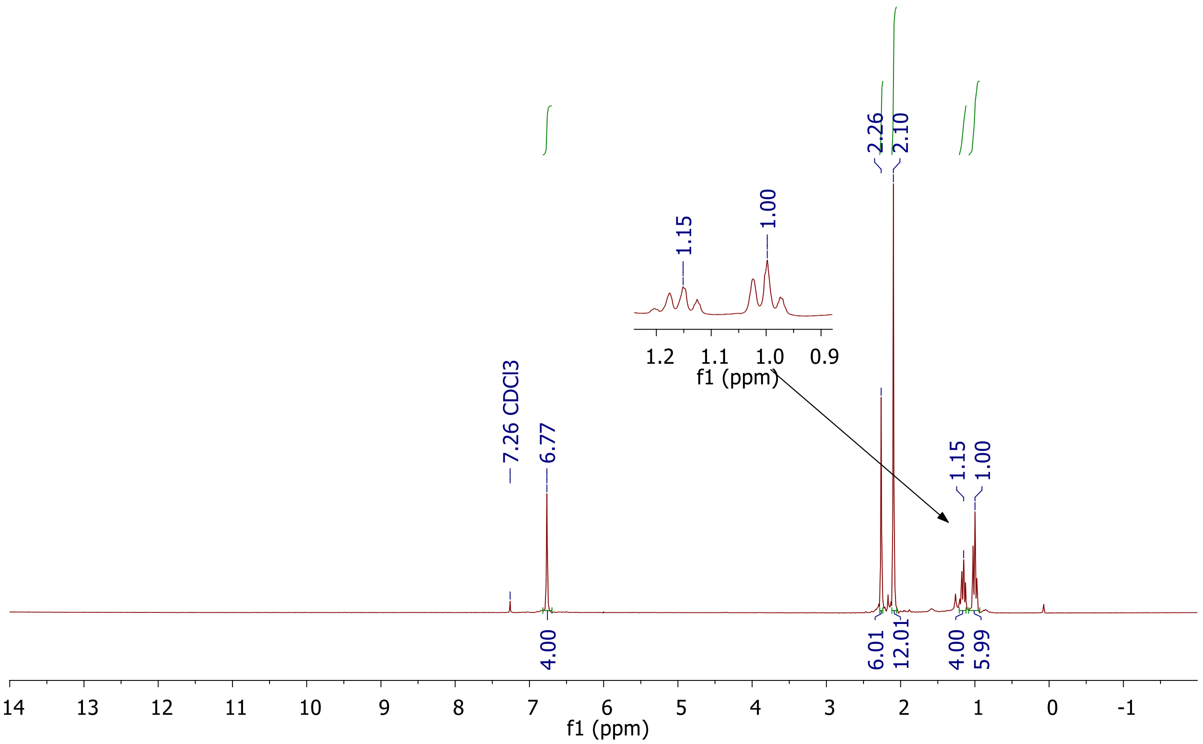

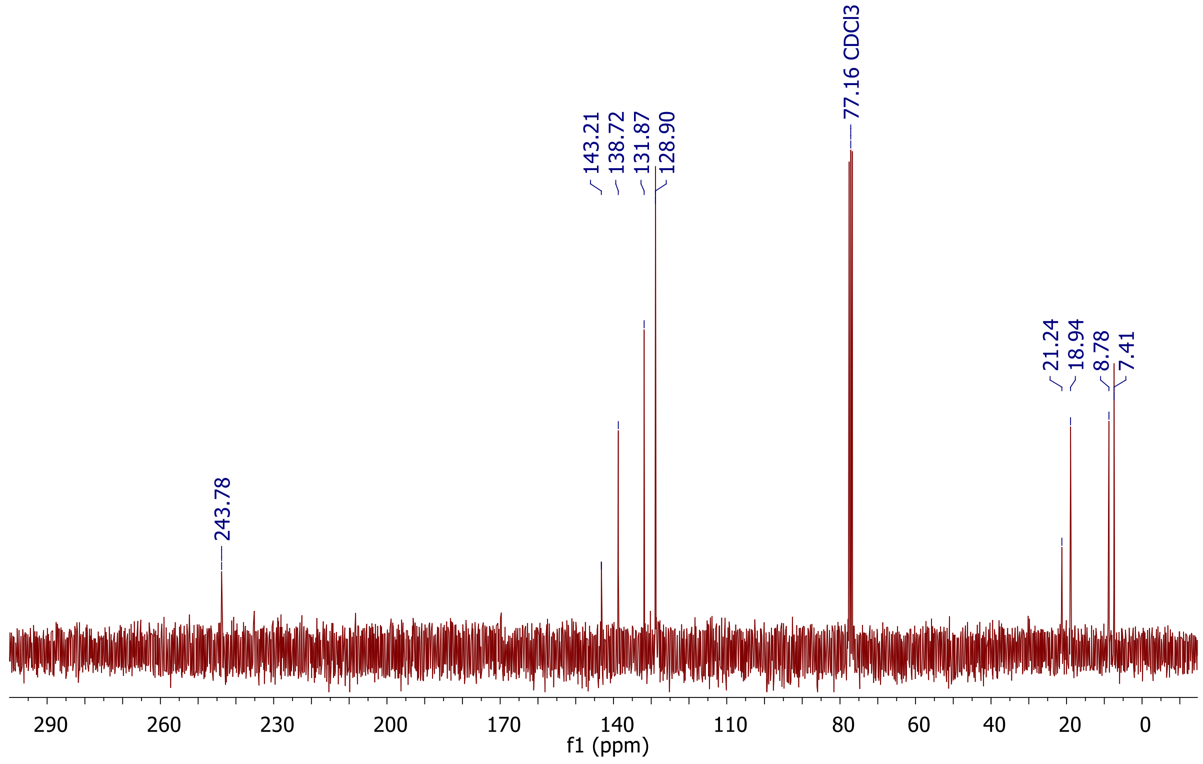

Figure S16: ^{1}H- and ^{13}C-NMR spectra of $(PhC(O))_2Ge(C(O)Mes)_2$ (**7c**) (C_6D_6 solution, vs ext. TMS, ppm)

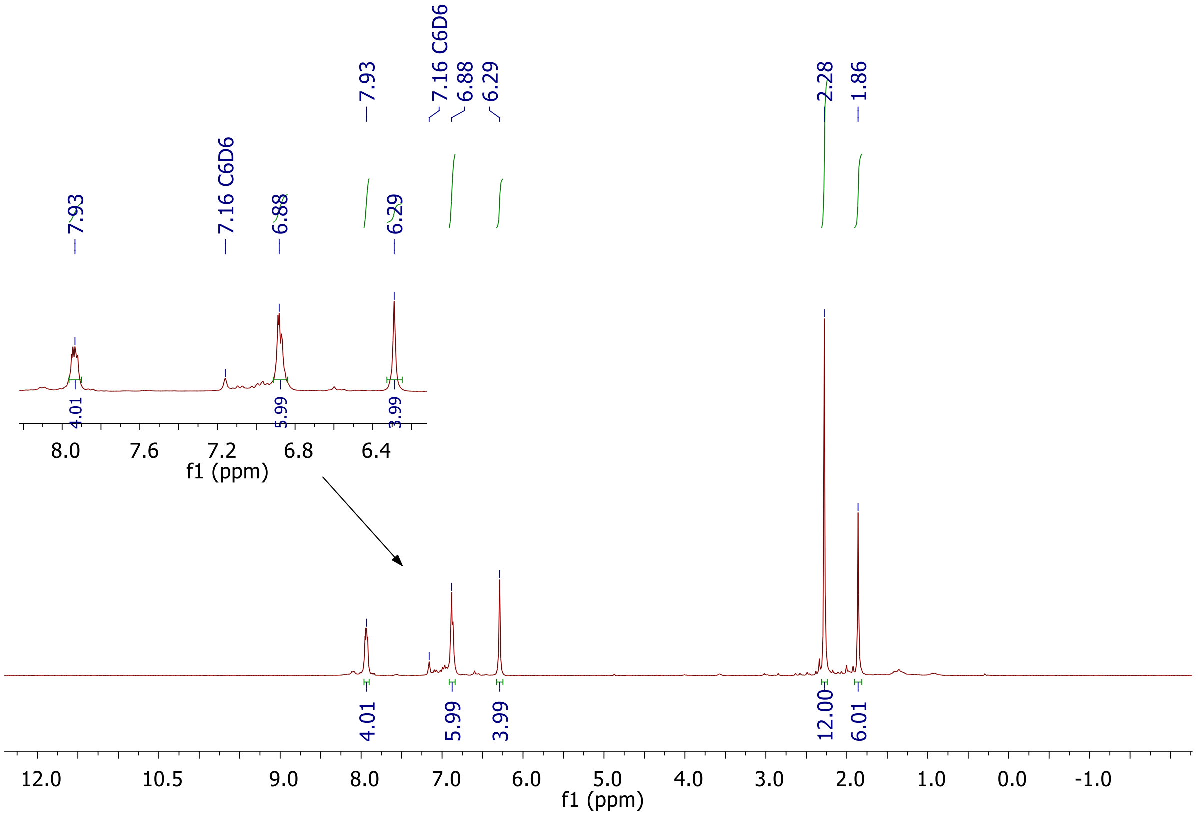

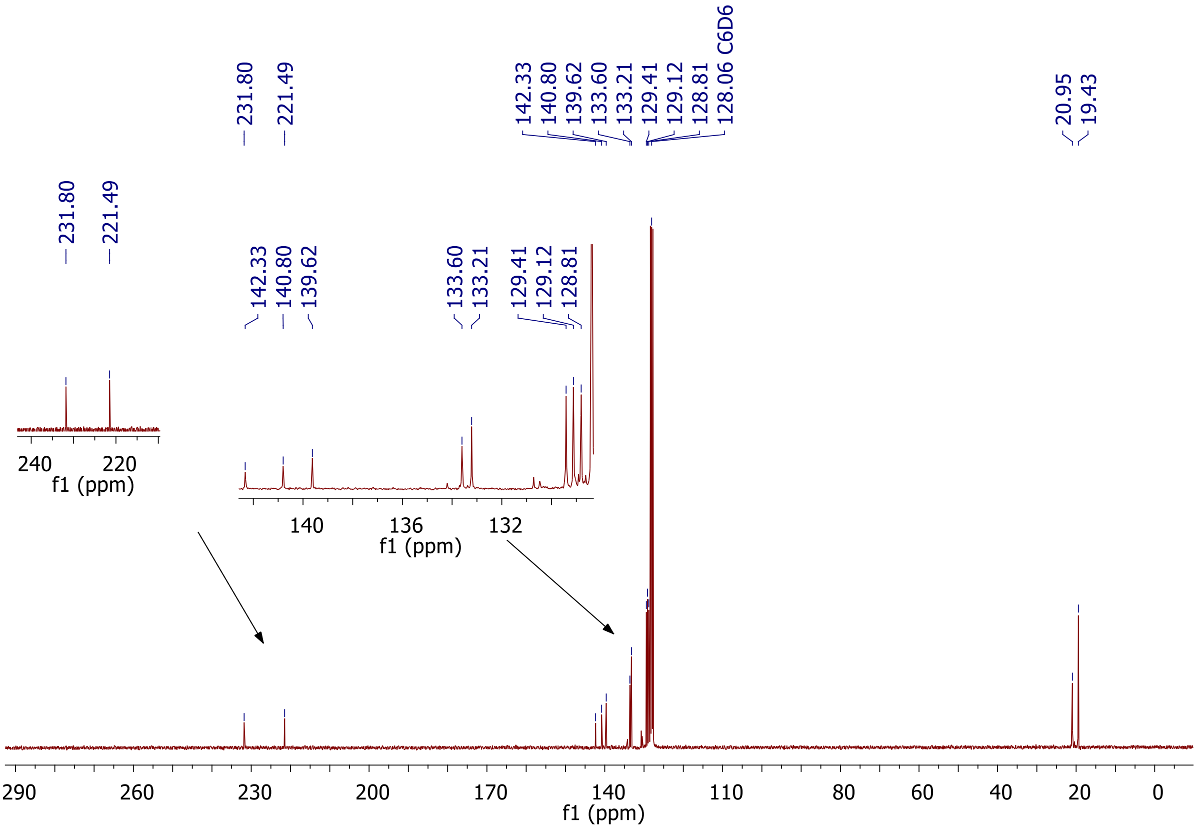

Figure S17: ^{1}H- and ^{13}C-NMR spectra of (*o*-TolC(O))$_2$Ge(C(O)Mes)$_2$ (**7d**) ($CDCl_3$ solution, vs ext. TMS, ppm)

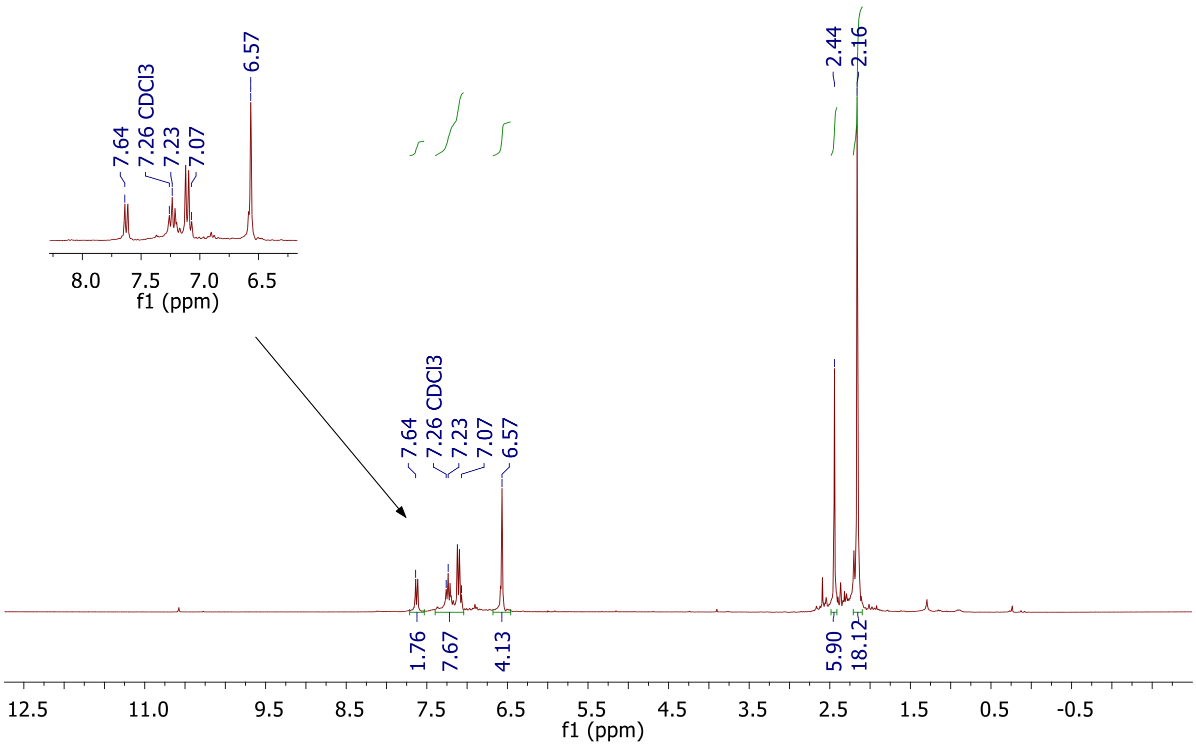

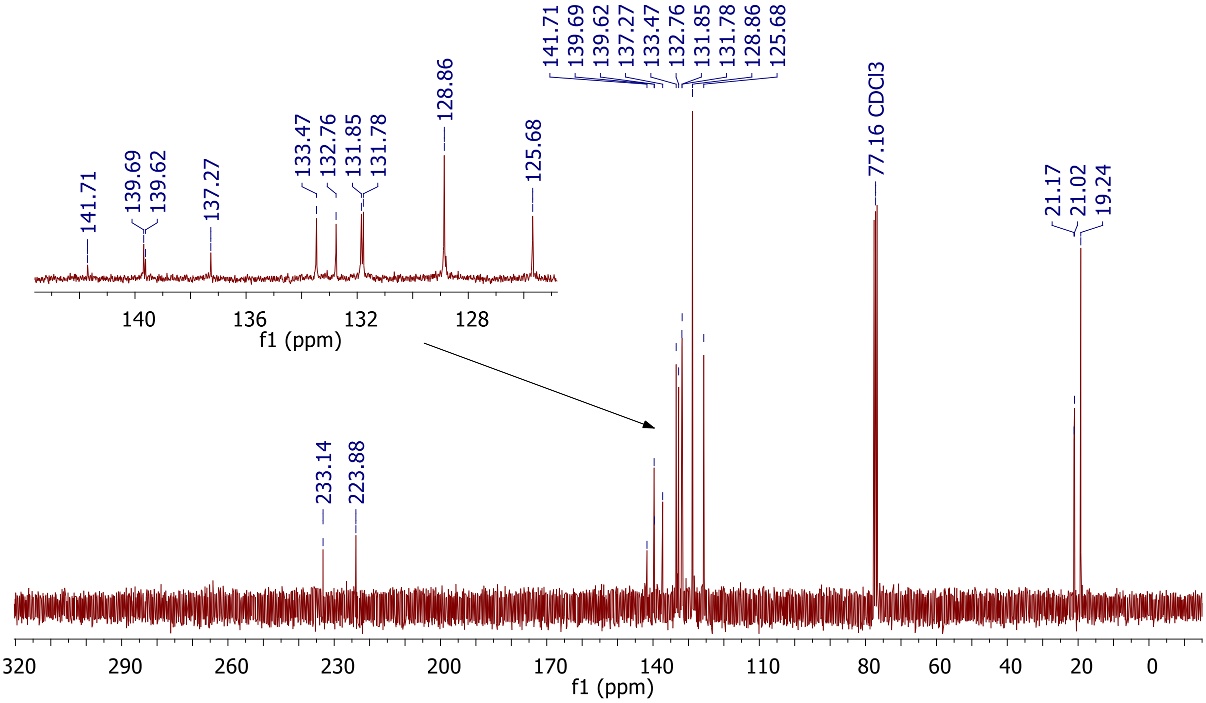

Figure S18: ^{1}H- and ^{13}C-NMR spectra of $(AdC(O))_2Ge(C(O)Mes)_2$ (**7e**) (C_6D_6 solution, vs ext. TMS, ppm)

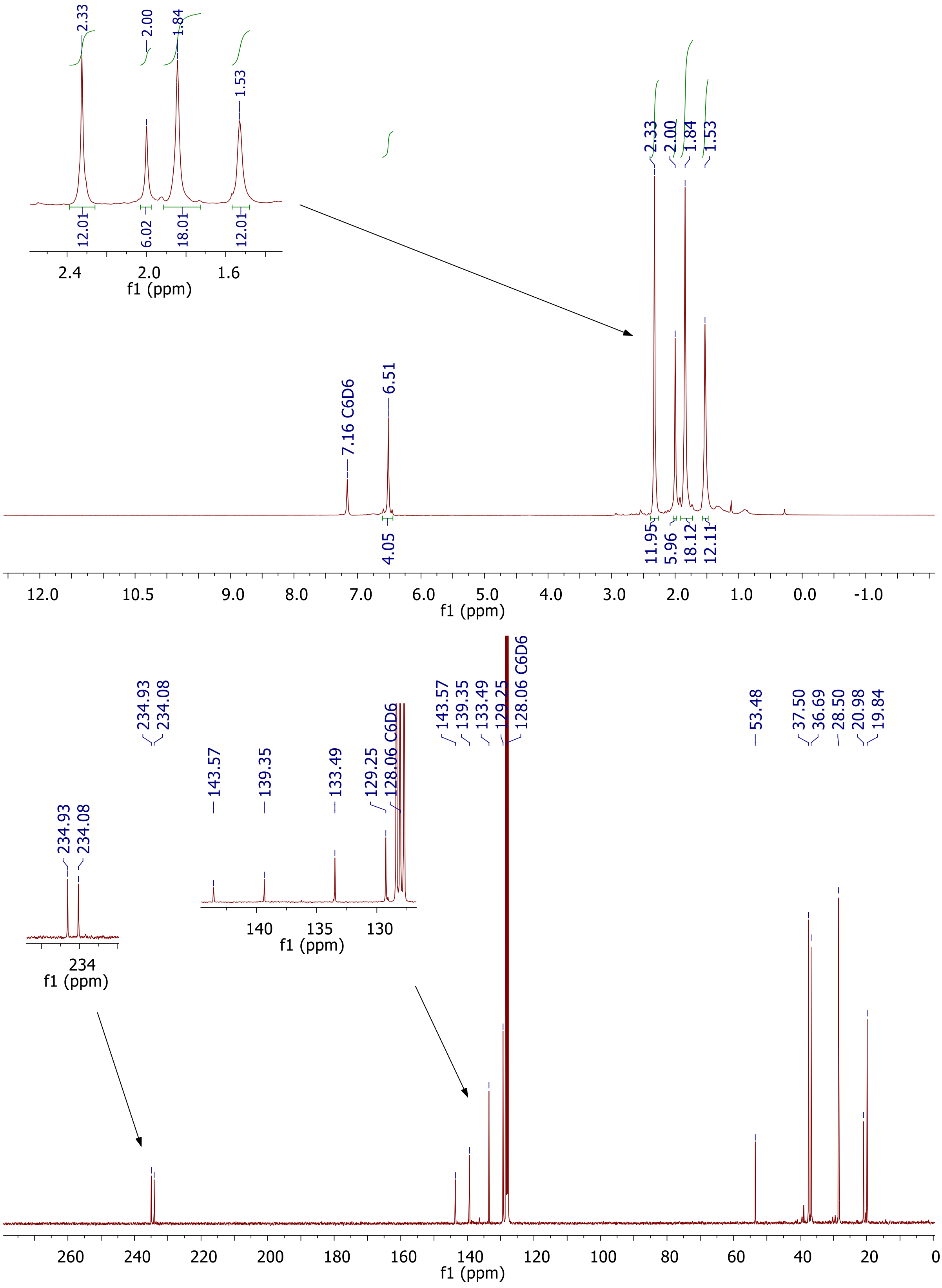

Figure S19: ^{1}H- and ^{13}C-NMR spectra of (tBuC(O))$_2$Ge(C(O)Mes)$_2$ (**7f**) (C_6D_6 solution, vs ext. TMS, ppm)

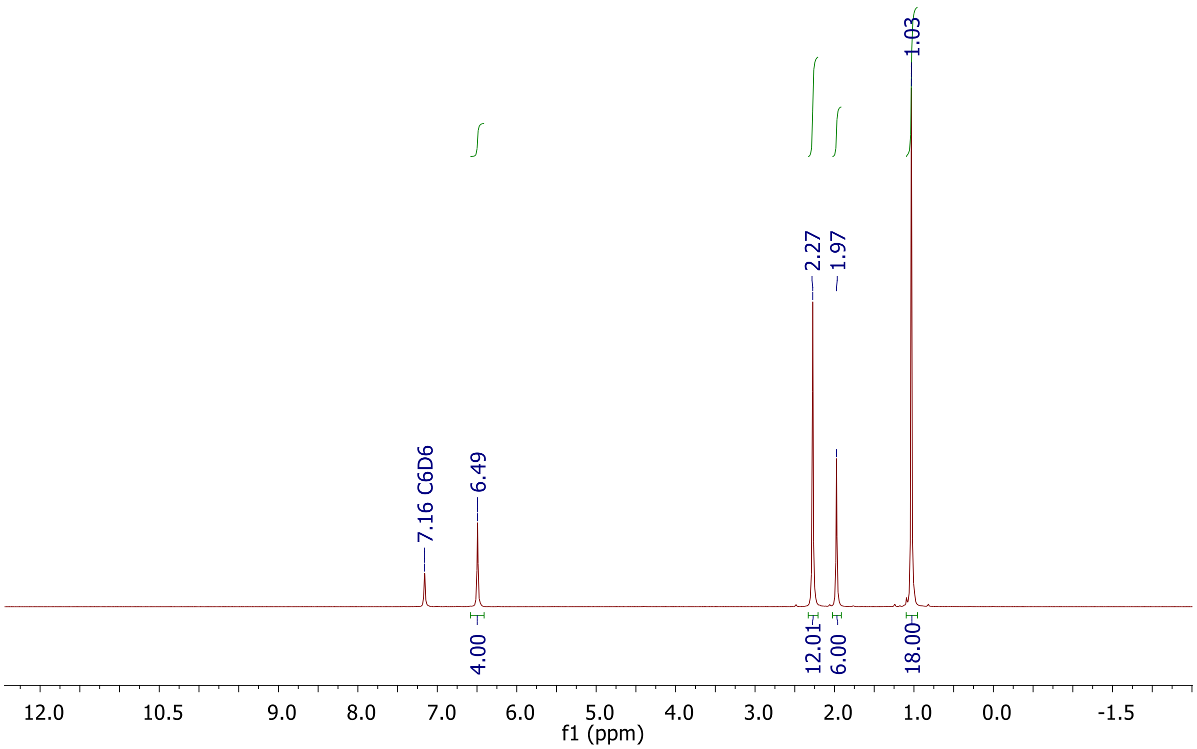

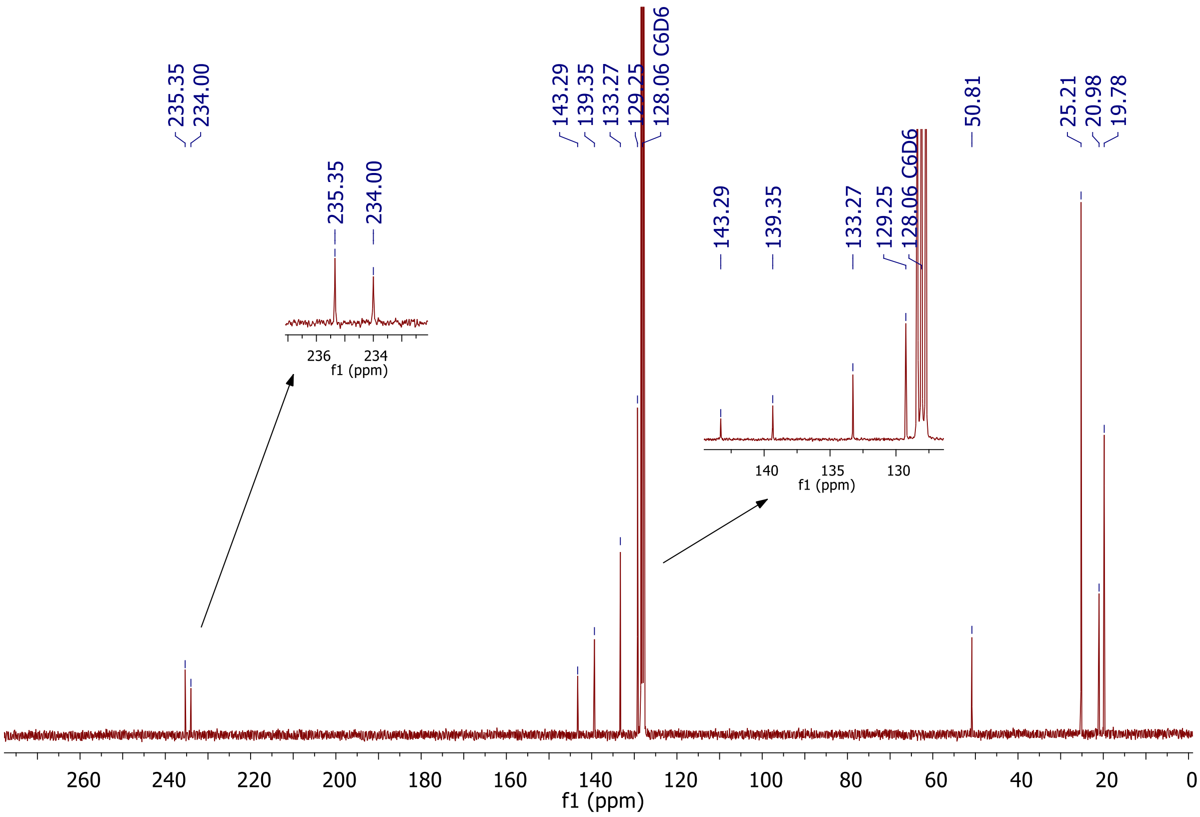

Figure S20: ^{1}H- and ^{13}C-NMR spectra of $(C_4H_8)Ge(C(O)Mes)_2$ (**7g**) (C_6D_6 solution, vs ext. TMS, ppm)

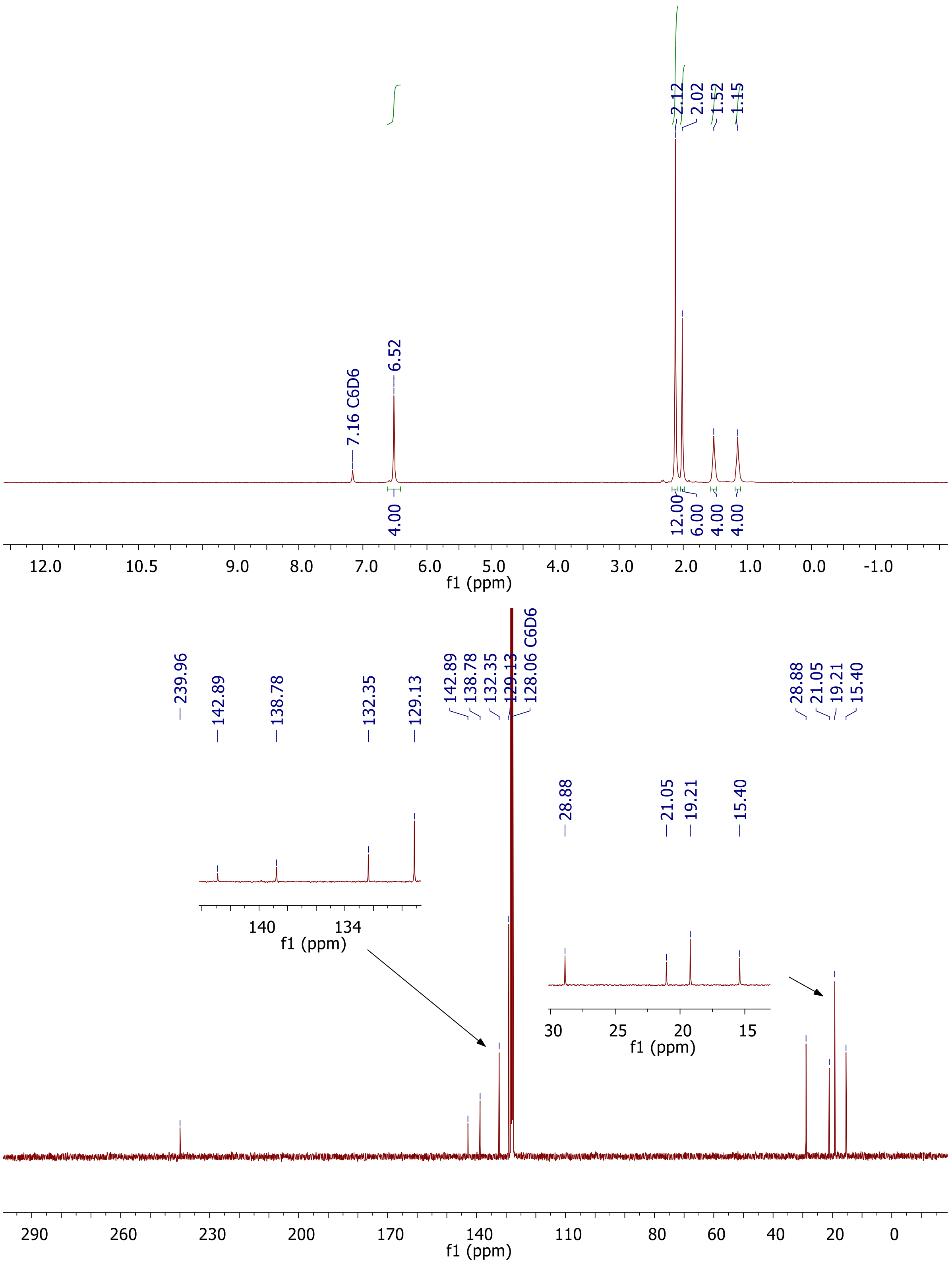

UV-Vis Spectroscopy

Figure S21: UV-Vis Spectrum of **2a** with three different concentrations. Measured in THF.

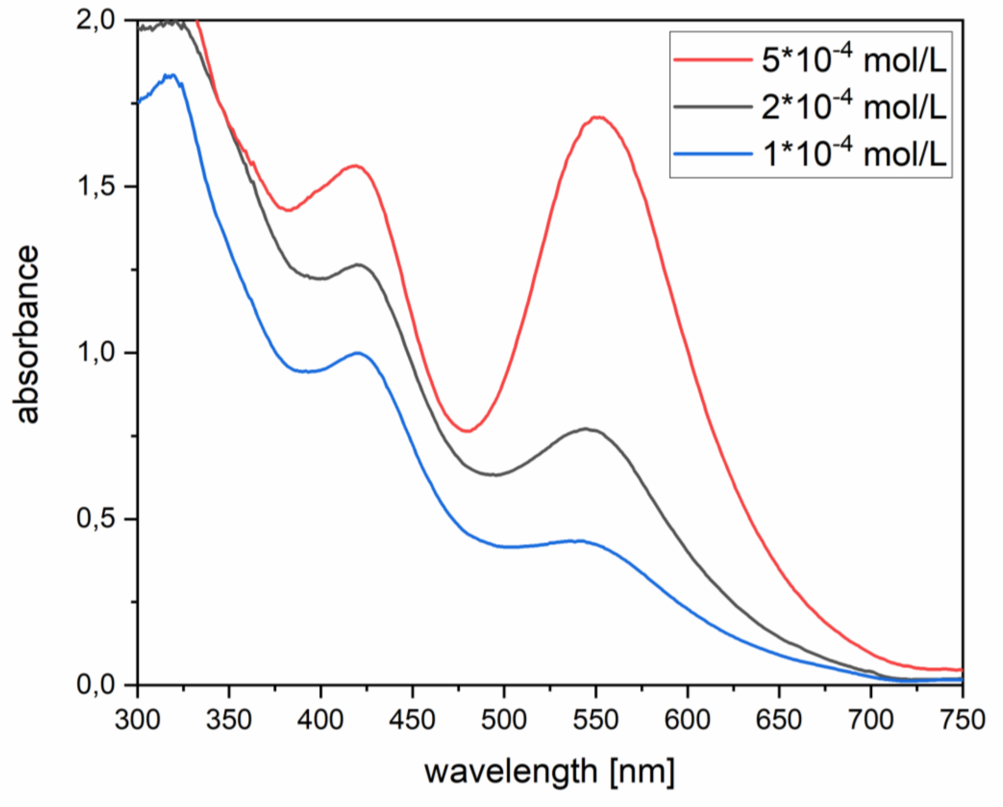

X-ray Crystallography

For single crystal X-ray diffractometry suitable crystals were covered with a layer of silicone oil. Under a microscope a single crystal was selected, mounted on a glass rod on a copper pin, and placed in the cold N_2 stream provided by an Oxford Cryosystems cryometer (T=100 K). XRD data collection was performed on a Bruker APEX II[14] diffractometer with use of Mo Kα radiation (λ= 0.71073 Å) from an IµS microsource and an APEX II CCD area detector. Data integration was carried out using SAINT.[14] Empirical absorption corrections were applied using SADABS. [15] The structure was solved by the dual space algorithm implemented in SHELXT.[16] Fourier analysis and refinement were performed by the full-matrix least-squares methods based on F^2 implemented in SHELXL[17] as implemented in SHELXLE.[18] The space group assignments and structural solutions were checked and evaluated using PLATON.[19] All non-hydrogen atoms were refined anisotropically. All hydrogen atoms were placed in calculated positions corresponding to standard bond lengths and angles using riding models. CIF files were edited, validated and formatted with the programs encifer[20] and Olex2.[21] Structural plots and figures were generated with MERCURY.[22]

Details on refinements of **2b**, **7b**, **7f** and **7g**:

2b cocrystallizes in the orthorhombic space group *Pnma* with two molecules of benzene per dianionic fragment. **7b** crystallizes in the orthorhombic space group *Pbca.* **7f** crystallizes in the monoclinic space group *C2/c.* **7g** crystallizes in the triclinic space group *P-1.* Details concerning data collection and refinement are provided in Crystallographic Table.

Although several samples were tested and recrystallization from other solvents was attempted, all crystals examined proved to be twinned. For integrations SAINT[14] was used and the data set was afterwards treated with TWINABS.[23] The structure was refined as a two-component twin against data in hkl5 format where the batch scale factor refined to a 51:49 ratio (twin law as determined by CELL_NOW[24]: 0.938 0.123 -0.047 / 0.976 -0.938 -0.025 / -0.05 0.04 -1.00)).

Crystallographic Table

Identification code	**7a**	**2b**	**7f**	**7g**
CCDC	2089190	2089189	2089191	2089192
Empirical formula	$C_{22}H_{28}GeO_2$	$C_{56}H_{82}GeK_2O_{14}$	$C_{30}H_{40}GeO_4$	$C_{24}H_{30}GeO_2$
Formula weight	397.05	1130.00	537.21	423.07
Temperature/K	100(2)	100.01	99.97	99.59
Crystal system	orthorhombic	orthorhombic	monoclinic	triclinic
Space group	Pbcn	Pnma	C2/c	P-1
a/Å	17.4081(7)	22.902(3)	16.7639(8)	7.6940(3)
b/Å	8.2634(4)	25.569(5)	13.1927(5)	9.3798(3)
c/Å	14.5356(6)	9.9717(12)	15.1283(7)	16.0408(6)
α/°	90	90	90	83.849(2)
β/°	90	90	123.262(2)	87.588(2)
γ/°	90	90	90	69.5880(10)
Volume/Å^3	2090.95(16)	5839.2(14)	2797.7(2)	1078.69(7)
Z	4	4	4	2
ρ_{calc}g/cm^3	1.261	1.285	1.275	1.303
μ/mm^{-1}	1.476	0.728	1.127	1.435
F(000)	832.0	2400.0	1136.0	444.0
Crystal size/mm^3	0.08×0.08×0.07	0.24×0.12×0.09	0.34×0.26×0.17	0.24×0.19×0.14
Radiation	MoK$_\alpha$ (λ = 0.71073)	MoK$_\alpha$ (λ = 0.71073)	MoK$_\alpha$ (λ = 0.71073)	MoK$_\alpha$ (λ = 0.71073)
2Θ range for data collection/°	4.68 to 66.538	4.384 to 55.99	4.24 to 60.37	5.086 to 57.482
Index ranges	$-26 \le h \le 26$<br>$-12 \le k \le 12$<br>$-22 \le l \le 22$	$-30 \le h \le 27$<br>$-29 \le k \le 33$<br>$-13 \le l \le 12$	$-23 \le h \le 23$<br>$-18 \le k \le 17$<br>$-21 \le l \le 21$	$-10 \le h \le 10$<br>$-12 \le k \le 12$<br>$0 \le l \le 21$
Reflections collected	178383	30840	31445	5594
Independent reflections	4008<br>R_{int} = 0.1083<br>R_{sigma} = 0.0277	7186<br>R_{int} = 0.0956,<br>R_{sigma} = 0.0994	4122<br>R_{int} = 0.0895,<br>R_{sigma} = 0.0613	5594<br>R_{sigma} = 0.0231
Data/restraints/parameters	4008/0/118	7186/0/343	4122/0/165	5594/0/251
Goodness-of-fit on F^2	1.083	1.033	1.032	1.043
Final R indexes [I>=2σ (I)]	R_1 = 0.0308,<br>wR_2 = 0.0732	R_1 = 0.0543,<br>wR_2 = 0.1241	R_1 = 0.0371,<br>wR_2 = 0.0869	R_1 = 0.0252,<br>wR_2 = 0.0609
Final R indexes [all data]	R_1 = 0.0516,<br>wR_2 = 0.0841	R_1 = 0.1107,<br>wR_2 = 0.1584	R_1 = 0.0469,<br>wR_2 = 0.0917	R_1 = 0.0292,<br>wR_2 = 0.0633
Largest diff. peak/hole / e Å^{-3}	0.55/-0.41	0.90/-1.07	0.89/-0.90	0.48/-0.49

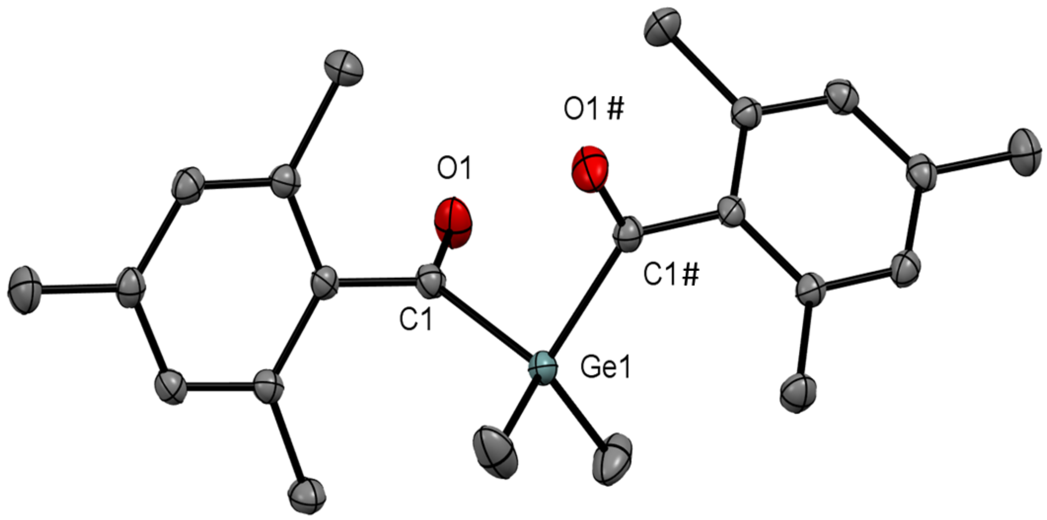

Figure S22: ORTEP representation for compound **7a**

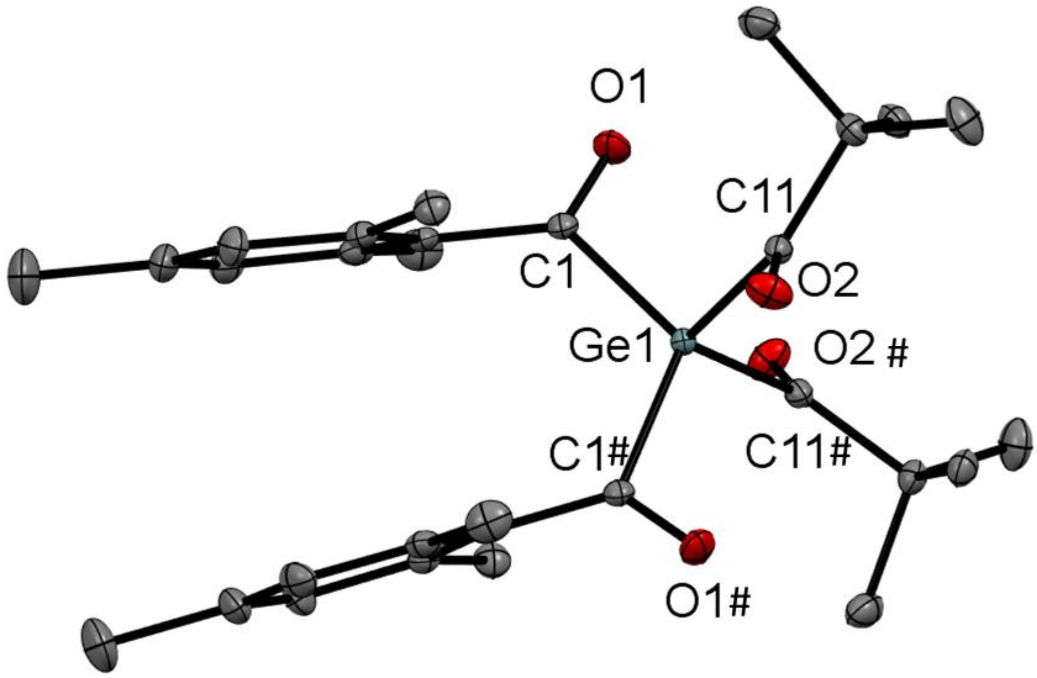

Figure S23: ORTEP representation for compound **7f**

References

[1] A. B. Pangborn, M. A. Giardello, R. H. Grubbs, R. K. Rosen, F. J. Timmers, *Organometallics* **1996**, *15*, 1518.
[2] A. G. Brook, F. Abdesaken, H. Söllradl, *Journal of Organometallic Chemistry* **1986**, *299*, 9.
[3] J. Radebner, A. Eibel, M. Leypold, C. Gorsche, L. Schuh, R. Fischer, A. Torvisco, D. Neshchadin, R. Geier, N. Moszner et al., *Angewandte Chemie (International ed. in English)* **2017**, *56*, 3103.
[4] a) A. D. Becke, *The Journal of Chemical Physics* **1993**, *98*, 5648; b) P. J. Stephens, F. J. Devlin, C. F. Chabalowski, M. J. Frisch, *J. Phys. Chem.* **1994**, *98*, 11623.
[5] a) S. Grimme, J. Antony, S. Ehrlich, H. Krieg, *The Journal of Chemical Physics* **2010**, *132*, 154104; b) S. Grimme, S. Ehrlich, L. Goerigk, *Journal of computational chemistry* **2011**, *32*, 1456.
[6] A. Schäfer, H. Horn, R. Ahlrichs, *The Journal of Chemical Physics* **1992**, *97*, 2571.
[7] F. Weigend, M. Häser, H. Patzelt, R. Ahlrichs, *Chemical Physics Letters* **1998**, *294*, 143.
[8] F. Neese, *WIREs Comput Mol Sci* **2012**, *2*, 73.
[9] M. Cossi, N. Rega, G. Scalmani, V. Barone, *The Journal of Chemical Physics* **2001**, *114*, 5691.
[10] D. R. Duling, *Journal of magnetic resonance. Series B* **1994**, *104*, 105.
[11] P. Frühwirt, A. Knoechl, M. Pillinger, S. M. Müller, P. T. Wasdin, R. C. Fischer, J. Radebner, A. Torvisco, N. Moszner, A.-M. Kelterer et al., *Inorganic chemistry* **2020**, *59*, 15204.
[12] M. Zalibera, P.-N. Stébé, K. Dietliker, H. Grützmacher, M. Spichty, G. Gescheidt, *Eur. J. Org. Chem.* **2014**, *2014*, 331.
[13] R. E. Taylor, *Concepts Magn. Reson.* **2004**, *22A*, 37.
[14] Bruker APEX2 and SAINT, Bruker AXS Inc.: Madison, Wisconsin, USA, 2012.
[15] a) R. H. Blessing, *Acta crystallographica. Section A, Foundations of crystallography* **1995**, *51 (Pt 1)*, 33; b) G. M. Sheldrick, SADABS, Univ. Göttingen, Ger. 1996.
[16] G. M. Sheldrick, *Acta crystallographica. Section A, Foundations and advances* **2015**, *71*, 3.
[17] G. M. Sheldrick, *Acta crystallographica. Section C, Structural chemistry* **2015**, *71*, 3.
[18] C. B. Hübschle, G. M. Sheldrick, B. Dittrich, *J Appl Cryst* **2011**, *44*, 1281.
[19] a) A. L. Spek, *Acta crystallographica. Section D, Biological crystallography* **2009**, *65*, 148; b) A. L. Spek, *J Appl Crystallogr* **2003**, *36*, 7.
[20] F. H. Allen, O. Johnson, G. P. Shields, B. R. Smith, M. Towler, *J Appl Cryst* **2004**, *37*, 335.
[21] O. V. Dolomanov, L. J. Bourhis, R. J. Gildea, J.A.K. Howard, H. Puschmann, *J Appl Cryst* **2009**, *42*, 339.
[22] C. F. Macrae, I. Sovago, S. J. Cottrell, P. T. A. Galek, P. McCabe, E. Pidcock, M. Platings, G. P. Shields, J. S. Stevens, M. Towler et al., *J Appl Cryst* **2020**, *53*, 226.
[23] G. M. Sheldrick, TWINABS, Univ. Göttingen, Ger. 1996.
[24] G.M. Sheldrick, CELL_NOW, Univ. Göttingen, Ger. 2008.
